# Optimizing *Escherichia coli* as a protein expression platform to produce *Mycobacterium tuberculosis* immunogenic proteins

**DOI:** 10.1186/1475-2859-12-115

**Published:** 2013-11-19

**Authors:** Luciano Piubelli, Manuela Campa, Caterina Temporini, Elisa Binda, Francesca Mangione, Massimo Amicosante, Marco Terreni, Flavia Marinelli, Loredano Pollegioni

**Affiliations:** 1 Department of Biotechnology and Life Sciences, University of Insubria, Varese, Italy; 2 The Protein Factory, Interuniversity Centre Politecnico di Milano, ICRM CNR Milano and University of Insubria, Milan, Italy; 3 Department of Drug Sciences and Italian Biocatalysis Centre, University of Pavia, Pavia, Italy; 4 Department of Infection Diseases, Fondazione IRCCS-Policlinico San Matteo, Pavia, Italy; 5 Department of Biomedicine and Prevention, University of Rome Tor Vergata, Rome, Italy; 6 ProxAgen Ltd., Sofia, Bulgaria; 7 Present address: Foundation Edmund Mach, San Michele all'Adige, Trento, Italy

**Keywords:** Recombinant antigens, *Mycobacterium tuberculosis*, Chimeric protein, Protein expression

## Abstract

**Background:**

A number of valuable candidates as tuberculosis vaccine have been reported, some of which have already entered clinical trials. The new vaccines, especially subunit vaccines, need multiple administrations in order to maintain adequate life-long immune memory: this demands for high production levels and degree of purity.

**Results:**

In this study, TB10.4, Ag85B and a TB10.4-Ag85B chimeric protein (here-after referred as *full*) - immunodominant antigens of *Mycobacterium tuberculosis* - were expressed in *Escherichia coli* and purified to homogeneity. The rational design of expression constructs and optimization of fermentation and purification conditions allowed a marked increase in solubility and yield of the recombinant antigens. Indeed, scaling up of the process guaranteed mass production of all these three antigens (2.5-25 mg of pure protein/L cultivation broth). Quality of produced soluble proteins was evaluated both by mass spectrometry to assess the purity of final preparations, and by circular dichroism spectroscopy to ascertain the protein conformation. Immunological tests of the different protein products demonstrated that when TB10.4 was fused to Ag85B, the chimeric protein was more immunoreactive than either of the immunogenic protein alone.

**Conclusions:**

We reached the goal of purifying large quantities of soluble antigens effective in generating immunological response against *M. tuberculosis* by a robust, controlled, scalable and economically feasible production process.

## Background

Tuberculosis (TB) is one of the leading cause of morbidity and mortality in humans, and it represents a major public health problem in many countries [1,2]. Despite *Mycobacterium tuberculosis,* the causative agent of TB, being identified by Robert Koch in 1882, major gaps remain in our knowledge of the complex cell life of this pathogen. Designing an effective vaccine against TB is an international research priority since human trials have demonstrated a highly variable protective efficacy of the currently used vaccine *Mycobacterium bovis* bacillus Calmette and Guerin (BCG) [2]. BCG is protecting against severe forms of childhood TB, but it is of limited use against adult pulmonary disease and its protective efficacy wanes significantly over a period of 10–15 years [3,4]. Due to the increasing incidence of multi- and extreme-drug-resistant *M. tuberculosis* clinical isolates, chemotherapeutic treatment options are often limited, toxic and of questionable efficacy. An improved second generation vaccine that can act as an efficient prophylactic vaccine and/or a vaccine that can boost immunity in BCG-vaccinated individuals is, therefore, urgently needed [5].

The availability of the *M. tuberculosis* genome sequence and the current efforts to sequence a large number of additional mycobacterial genomes [5] have set the stage for a post-genomics approach to the identification, production and trials of novel antigens. Gene families of immunodominant proteins have been identified *in silico* and tested *in vivo*. Among them, Ag85 complex (Ag85 A, B, C), the most abundant protein secreted by *M. tuberculosis*, attracts considerable interest for a new TB vaccine development. Ag85B, a mycolyl transferase, is among the most potent antigen species yet identified, which induces both humoral and cell-mediated immune response in *M. tuberculosis*-infected subjects [6,7]. Another gene family, the *esat-6* one, has been demonstrated to encode several immunodominant proteins that are strongly recognized by the immune systems of different animal models of TB, as well as by T cells from human beings exposed to *M. tuberculosis *[8]. TB10.4 is a low molecular mass protein belonging to *esat-6* family. This protein represents a valuable vaccine candidate since it is highly conserved in different *M. tuberculosis* clinical isolates obtained from different geographical locations and it is strongly recognized by T-cells from BCG-vaccinate donors as well as from TB patients [8]. The subunit vaccine composed of Ag85B-TB10.4 has been also designed and shown to generate a strong, specific immune response in mice. Vaccinating mice with this subunit vaccine induced a level of protection against TB comparable to that produced by BCG and better than that achieved by vaccinating with either of the single proteins [6].

To support further evaluation of *M. tuberculosis* selected antigens and their modified recombinant variants as candidates for the second-generation vaccines against TB, a reliable and robust production process is urgently needed today. Vaccine candidates should be produced in the quantity and quality needed for preclinical and clinical studies. Although it is possible to purify native antigens from *M. tuberculosis,* it is more efficient and safer to express them in an heterologous host such as *Escherichia coli* and then optimize the recombinant protein production process [9,10]. With this approach, molecular engineering may be used to redesign antigens of interest and to combine and fuse two antigens such in the case of the Ag85B-TB10.4 subunit vaccine. Unfortunately until now most of the recombinant antigens currently explored as TB vaccine candidates have been purified in very low amount from *E. coli,* making the production process for large scale use extremely costly. Due to the tendency of incorrect protein folding and aggregation when over-expressed in an heterologous system, these antigens often accumulated in inclusion bodies (IBs), from whom they were recovered by solubilisation with denaturant agents followed by tailored procedures of protein renaturation [11,12]. The use and the following removal of large amount of denaturant agents increase the production cost and require additional steps of quality control. In addition, recent data demonstrated that *M. tuberculosis* antigens purified from IBs do not retain the conformation adopted by the soluble counterparts, raising the question of whether these recombinant proteins keep the same immunogenicity of the native antigens [12].

In this paper, we have adopted different strategies to express recombinant Ag85B, TB10.4 and the fused TB10.4-Ag85B chimeric protein, with the aim of purifying these antigens in a soluble form from *E. coli* cells. Chemical identity and stability, immunological activity and, last but not least, production feasibility and cost of the produced recombinant proteins have been assessed to sustain their further development as vaccine candidates *per se* or as a scaffold for further structural modifications. Production and purification processes of these immunogenic proteins have been successfully scaled up from shaken flasks to bench bioreactors.

## Results

### Production and purification of Trx-TB10.4 and TB10.4

**Figure 1:**
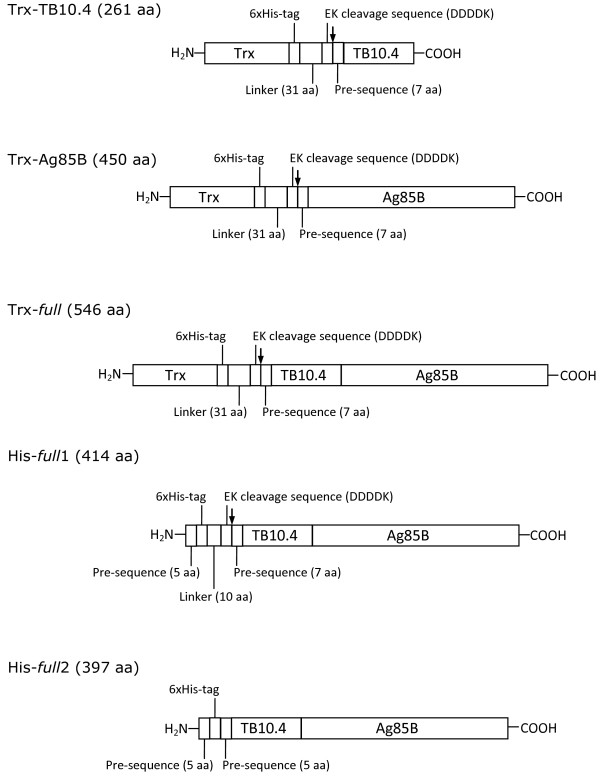
**Scheme of the recombinant proteins as produced upon cloning in pET32b (Trx-TB10.4, Trx-Ag85B and Trx- *full *) or pColdI (His- *full *1 and His- *full *2) vectors.** Trx: thioredoxin (116 amino acids, aa). The arrow indicates the EK cleavage site. The entire amino acid sequences are reported in the Additional file 1: Figure S2.

**Figure 2:**
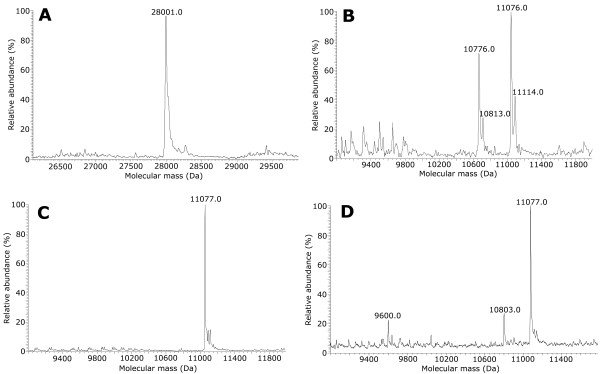
**MS analyses of purified Trx-TB10.4 and TB10.4.** Deconvoluted spectra from intact ESI-LIT-MS analyses of: **A)** Trx-TB10.4; **B)** purified TB10.4 before the proteolytic cleavage optimization; **C)** purified TB10.4 after the proteolytic cleavage optimization and **D)** the same sample after further incubation at 37°C, for 24 hours.

The synthetic cDNA coding for TB10.4 (GenBank Accession no. CAA17363.1) was optimized to match the codon usage for *E. coli* expression (see Additional file 1: Figures S1 and S2). Codon usage optimization was essential since *E. coli* is a low G + C content (~ 50%) Gram-negative bacterium while *M. tuberculosis* is a high G + C (> 65%) Gram-positive actinobacterium. The cDNA was subcloned into pET32b plasmid in frame with nucleotidic sequence coding for thioredoxin (Trx), with a His_6_-tag sequence and with a sequence recognized by enterokinase (EK) – these sequences are located at the N-terminus of TB10.4 (Figure 1). Trx is a 12 kDa protein that remarkably increases the solubility of fusion proteins [13]. The chimeric Trx-TB10.4 protein (261 amino acids, molecular mass 28.1 kDa, Table 1 and Figure 1) was produced in *E. coli* using BL21(DE3) strains. Basal expression was performed in LB medium, adding 0.1 mM IPTG when OD_600nm_ reached 0.6-0.8 and collecting cells after additional 4 hours of growth at 18°C: using these conditions, a fairly good amount of chimeric protein was produced as soluble form and about 2 mg/L of > 85% pure Trx-TB10.4 was purified after a single-step purification on nickel-affinity chelating column. Optimization of the expression conditions (i.e. growing cells for 16 hours at 18°C after IPTG addition) increased significantly the production of the soluble form of the chimeric protein: 12 mg/L of > 95% pure Trx-TB10.4 was purified by metal-chelating chromatography (Table 2, see Additional file 2: Figures S3A, B and C). Mass spectrometry (MS) spectrum of the fusion protein reported in Figure 2A confirmed the identity and purity of the fused protein, indicating a molecular mass corresponding to the isoform lacking methionine at the N-terminal.

**Table 1:** Chemico-physical parameters of the recombinant proteins as produced upon cloning in pET32b or pColdI

**Protein**	**Length (aa)**	**Molecular mass (Da)**	**Theoretical pI**
Trx-TB10.4	261	28,133.6	5.06
TB10.4	103	11,076.3	4.44
Trx-Ag85B	450	48,405.2	5.11
Ag85B	292	31,347.9	4.77
Trx-*full*	546	58,777.7	4.99
His-*full*1	414	44,865.9	5.11
*full*1	388	41,720.5	4.71
His-*full*2	397	43,060.0	5.53

Theoretical isoelectric point (pI) was calculated by ExPASyBioinformatic Resource Portal (http://www.expasy.org/) using ProtParam tool.

**Table 2:** **Production yield of purified recombinant Trx-TB10.4, Trx-Ag85B (chimeric and single antigens, after proteolytic cleavage), and His- *full *** **2 by *E. coli *** **cells grown at flask and bioreactor scales**

**Protein**	**Growth conditions**	**Biomass at the harvest time**^**a **^**(g/L)**	**Pure chimeric protein**^**b**^** (mg/L)**	**Pure mature protein (mg/L)**
Trx-TB10.4	Flasks	4.8	12	4^c^
	Bioreactor	8.5	80	25^c^
Trx-Ag85B	Flasks	10.5	9	4.7^c^
	Bioreactor	19.5	30	16^c^
His-*full*2	Flasks	6.5	/	0.5
	Bioreactor	6.5	/	2.5

^a^grams of *E. coli* cells (wet weight) per liter of cultivation broth at the harvest time.

^b^milligrams of protein obtained upon purification by nickel-affinity chromatography per liter of cultivation broth.

^c^milligrams of protein obtained upon proteolytic cleavage and separation by nickel-affinity chromatography per liter of cultivation broth.

Complete cleavage of Trx-TB10.4 fusion protein was obtained using 3 U of recombinant enterokinase (rEK) per mg at 20°C for 16 hours. The cleaved products (Trx and TB10.4) were separated by metal-chelating chromatography (see Additional file 2: Figure S3D and E). The expected mature recombinant TB10.4 is a 103 amino acid long protein (Table 1), bearing a seven amino acid residue pre-sequence (AMAISDP) at the N-terminal region originating from the cloning procedure (Figure 1). MS analysis of TB10.4 preparation (Figure 2B) showed the presence of the full-length TB10.4, with an average mass of 11076.0 Da (nominal mass 11076.36 Da), and an additional product (accounting for *ca.* 40% of the total protein) with a calculated average mass of 10776.0 Da. This mass shift (-300 Da) corresponds to the lack of three C-terminal amino acids (WGG) from the mature TB10.4. This secondary cleavage might be due to the AEAAK sequence – resembling the EK consensus sequence (i.e. DDDDK) – that is located just before the three C-terminal amino acids. When the TB10.4 preparation was further incubated for 24 hours at 37°C, additional degradation products appeared, whose formation was instead avoided adding a specific EK inhibitor: this result indicates a persisting proteolytic activity by residual EK not (completely) removed by the chromatography on HiTrap chelating column following the cleavage step (see above). Formation of the truncated form was avoided using an optimized rEK (Tag · Off High Activity rEK) – 2 U per mg of substrate protein, 6 hours of incubation at 25°C – and removing rEK using EKapture Agarose (Figure 2C). Pure mature TB10.4 was recovered (Table 2) and its stability at room temperature was checked by MS analysis: following the optimized cleavage procedure, no secondary (truncated) forms were apparent. When the same preparation was further incubated for 24 hours at 37°C in the absence of an EK inhibitor *ca.* 74% of the mature TB10.4 form was still present (Figure 2D).

### Production and purification of Trx-Ag85B and Ag85B

**Figure 3:**
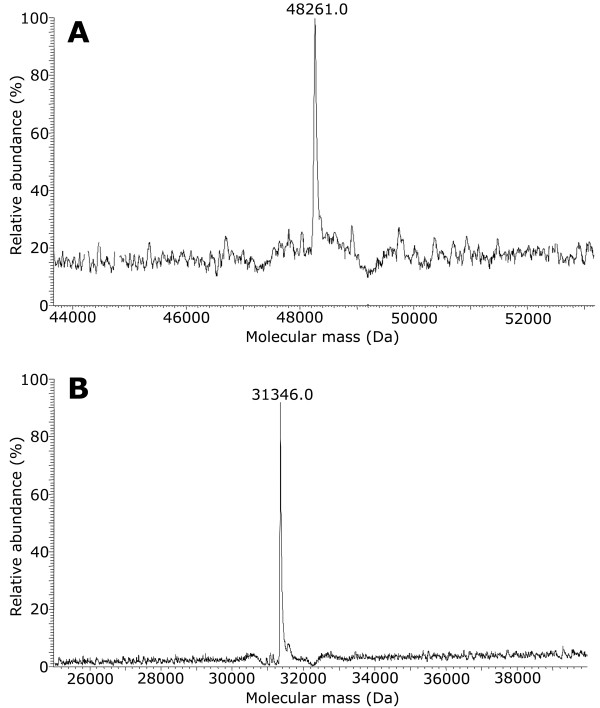
**MS analyses of purified Trx-Ag85B and Ag85B.** Deconvoluted spectra from intact ESI-LIT-MS analyses of: **A)** purified Trx-Ag85B; **B)** mature Ag85B after proteolytic cleavage.

The synthetic cDNA coding for the mature form (285 amino acids) of Ag85B lacking the region encoding for the N-terminal 40 residues corresponding to the putative signal peptide (UniProtKB/Swiss-Prot Accession no. P0C5B9.1; PDB Accession no. 1F0N_A), also optimized for *E. coli* expression, was subcloned into pET32b plasmid, in frame with nucleotidic sequence coding for Trx, with a His_6_-tag sequence and with a sequence recognized by EK – these sequences are located at the N-terminus of Ag85B (Figure 1). The Trx-Ag85B chimeric protein is 450 amino acid long and its molecular mass is 48.4 kDa (Table 1 and Figure 1, see also Additional file 1: Figures S1 and S2). Using the same standard conditions set up for Trx-TB10.4, a very low amount of soluble Trx-Ag85B was produced and a low amount of pure protein (< 1 mg/L) was isolated by metal chelating chromatography. In order to improve the amount of protein produced, a number of expression conditions - using different growth media (LB, TB, SB and minimal medium M9), adding 0.1 or 1.0 mM IPTG when OD_600nm_ reached 0.7, 2 or after overnight growth (saturation condition) - were tested. In all cases, following IPTG addition, cells were grown at 18°C and collected after 4 hours, and the expression level was assessed by Western blot using anti-His-tag antibody. The largest amount of soluble Trx-Ag85B was obtained growing cells in SB medium and inducing expression with 0.1 mM IPTG at an OD_600nm_ = 2 (see Additional file 2: Figure S4). Further improvement was obtained growing cells for additional 16 hours (overnight) after IPTG addition. Using these conditions, *ca*. 25-30% of the chimeric protein was soluble (Additional file 2: Figure S5B and C) corresponding to a productivity of 20 mg/L: 9 mg/L of Trx-Ag85B (> 95% purity degree) were isolated after a single-step purification on nickel-affinity column chromatography (Table 2, see Additional file 2: Figure S5A, B and C). MS spectrum of the fusion protein confirmed its identity and purity (Figure 3A).

Complete maturation of the fusion protein was obtained using 3 U of rEK per mg of Trx-Ag85B at 20°C for 16 hours (Table 2, see Additional file 2: Figure S5D and E). The mature Ag85B obtained by rEK cleavage is a 292 amino acid long protein (molecular mass: 31.3 kDa, see Table 1 and Figure 1), bearing at the N-terminal end a pre-sequence (AMAISDP) originating from the cloning procedure. MS analysis confirmed the identity and purity of the mature Ag85B, with a determined mass of 31346.0 Da (nominal mass 31347.9 Da, Figure 3B).

### Production and purification of TB10.4-Ag85B (*full*) protein

The synthetic cDNA coding for the fusion protein obtained by the combination of the nucleotide sequences of TB10.4 and Ag85B (namely, *full* protein) was subcloned into pET32b plasmid, in frame with the sequence coding for Trx, with a His_6_-tag sequence and with a sequence recognized by rEK (useful to remove Trx). The chimeric protein Trx-TB10.4-Ag85B (Trx-*full*) is 546 amino acid long (molecular mass is 58.8 kDa, Table 1 and Figure 1). Since the aim of this work was to produce soluble antigens, the TB10.4-Ag85B fusion protein was preferred to the Ag85B-TB10.4 version because the latter chimera was never produced in a significant amount as soluble protein [6,12]. The solubility of Trx-TB10.4-Ag85B protein was reported to be >10-fold increased compared to that of Trx-Ag85B-TB10.4 [12].

Trials for the expression of Trx-*full* were conducted using the same conditions set up for the single antigenic proteins. Western blot analysis of the produced Trx-*full* in cell extracts showed that a consistent fraction (80-90%) of the fusion protein accumulated into the insoluble fraction, being only traces of the protein detectable in the soluble fraction (and together with proteolytic products, data not shown). Co-expression of different chaperon proteins (AraB/DnaK/DnaJ/GrpE or GroES/GroEL/TiG) did not increase the solubility of the fusion protein. Purification of soluble Trx-*full* yielded less than 1 mg/L, with purity inferior to 50% (data not shown).

To obtain higher amounts of the *full* protein as soluble form, the chimeric cDNA was inserted into pColdI plasmid, a system based on low-temperature expression gene (cold shock gene) specifically designed to improve the solubility in *E. coli* of heterologous proteins [14]. Two different gene constructs were prepared, named His-*full*1 and His-*full*2: in both cases, the *full* protein bears the His-tag at the N-terminus without thioredoxin (Figure 1). His-*full*1 is a 414 amino acid long protein (molecular mass of 44.9 kDa, Table 1 and Figure 1, see also Additional file 1: Figure S2), still bearing at the N-terminal region the sequence recognized by EK. Expression trials for His-*full*1 were conducted in BL21(DE3) and BL21(DE3)pLysS *E. coli* strains, growing cells in LB, TB or SB media at 37°C, adding 1.0 mM IPTG when cells reached early/middle exponential growth phase (OD_600 nm_ = 0.6-0.7 for LB and 1.2-1.5 for TB and SB) or middle/late exponential growth phase (OD_600 nm_ = 1.2-1.5 for LB and 4–5 for TB and SB). Cells were then grown for additional 20 hours at 15°C prior harvesting. Western blot analysis of cells extracts showed the major part of the expressed His-*full*1 (*ca*. 80%) accumulated as IBs. A maximum yield of 1.3 mg/L of His-*full*1 with *ca.* 85% purity was obtained after a single-step purification (on HiTrap Chelating chromatography) from cells grown in TB medium added with 5 g/L of NaCl and by adding further 25 g/L of NaCl at the moment of induction with IPTG, according to a protocol previously developed in our lab [15] (data not shown). Incubation of purified His-*full*1 with EK in the same condition used for Trx-Ag85B (3 U/mg His-*full*1, for 16 hours at 20°C) yielded a consistent amount of proteolytic products, indicating a high sensitivity to proteolysis of the chimeric protein (not shown).

In order to eliminate the EK cleavage step and to reduce the length of the additional sequence at the N-terminal end of the chimeric protein, site-directed mutagenesis of the *full* cDNA introduced a *Nde*I restriction site allowing its cloning in pColdI plasmid using *Nde*I and *Eco*RI restriction sites (Figure 1). By this strategy, a 397 amino acid long protein (namely His-*full*2, molecular mass: 43.06 kDa, Table 1 and Figure 1) was designed. His-*full*2 contains 16 additional amino acids including the His_6_-tag sequence (MNHKVHHHHHHIEGRH, see Additional file 2: Figure S2) at the N-terminal end vs. the 33 amino acids present in the previous TB10.4-Ag85B chimeric proteins.

**Figure 4:**
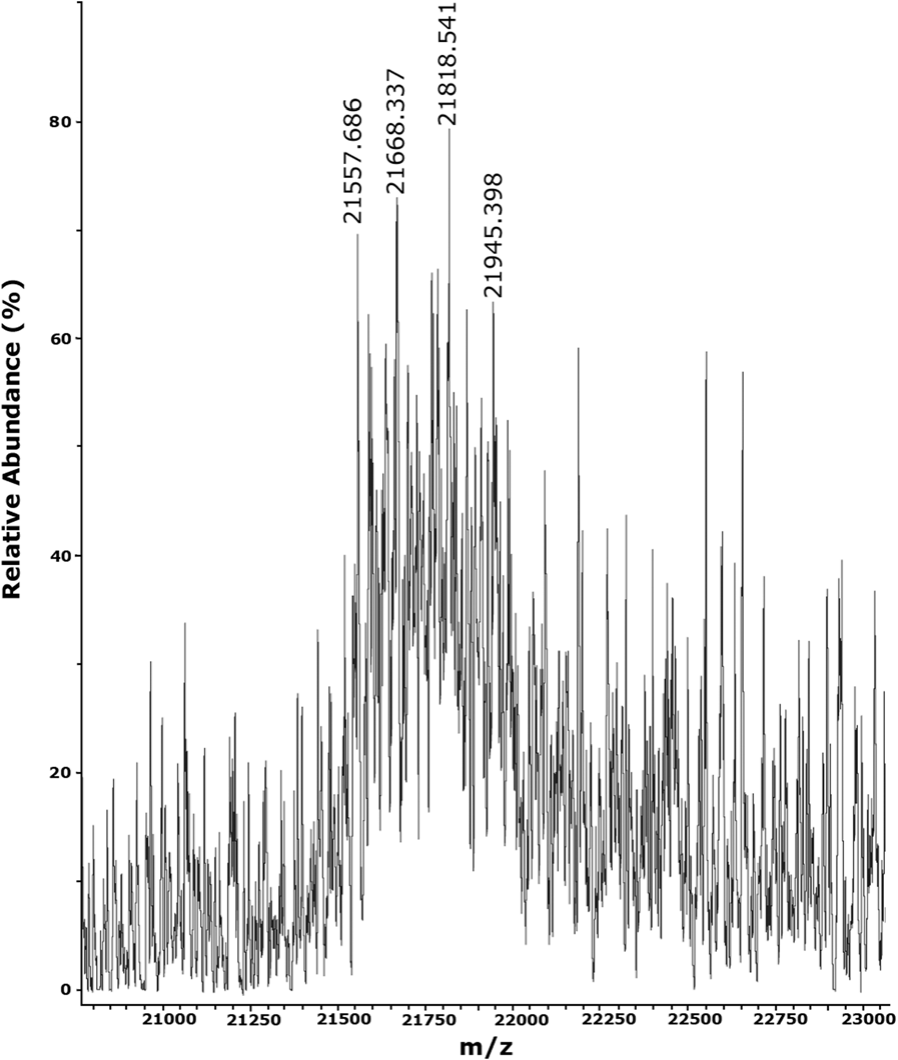
**MALDI-TOF/TOF mass spectrum of the doubly-charged protein His-*full2*.** Two principal isoforms can be observed, corresponding to the natrium adduct (at 21557.68 m/z) and to the natrium/synapinic acid adduct (21668.3 m/z).

Expression trials carried out on His-*full*2 showed that, similarly to Trx-Ag85B (see the paragraph above), the best expression conditions were based on the use of SB medium and induction with 0.1 mM IPTG at OD_600nm_ = 2. An increase in the production of the recombinant protein was obtained using NaCl in the medium (10 g/L) and by adding further 25 g/L of NaCl simultaneously to IPTG (not shown). As reported in Table 2 and explained below, His-*full*2 production increased significantly when scaled-up to a 3 L bioreactor. However, the major part of the fusion protein again accumulated as IBs (see Additional file 2: Figure S6B and C, that refer to cells produced at bioreactor scale). About 0.5 mg/L of His-*full*2 with a purity of *ca.* 90% was obtained after the HiTrap Chelating chromatography performed under optimized conditions (linear gradient from 100 to 250 mM imidazole in 3-column volumes, see Additional file 2: Figure S6A and C). Despite the lower yield than for the His-*full*1, this second chimeric protein had the advantage to skip the EK cleavage step. MALDI-TOF/TOF MS spectrum of the chimeric protein is given in Figure 4. The two principal ions observed correspond to the doubly charged natrium adduct (at 21557.68 *m/z*) and to the doubly charged natrium/synapinic acid adduct (21668.3 *m/z*) of the His-*full*2 protein, confirming its identity.

### Scaling up of protein antigen production

**Figure 5:**
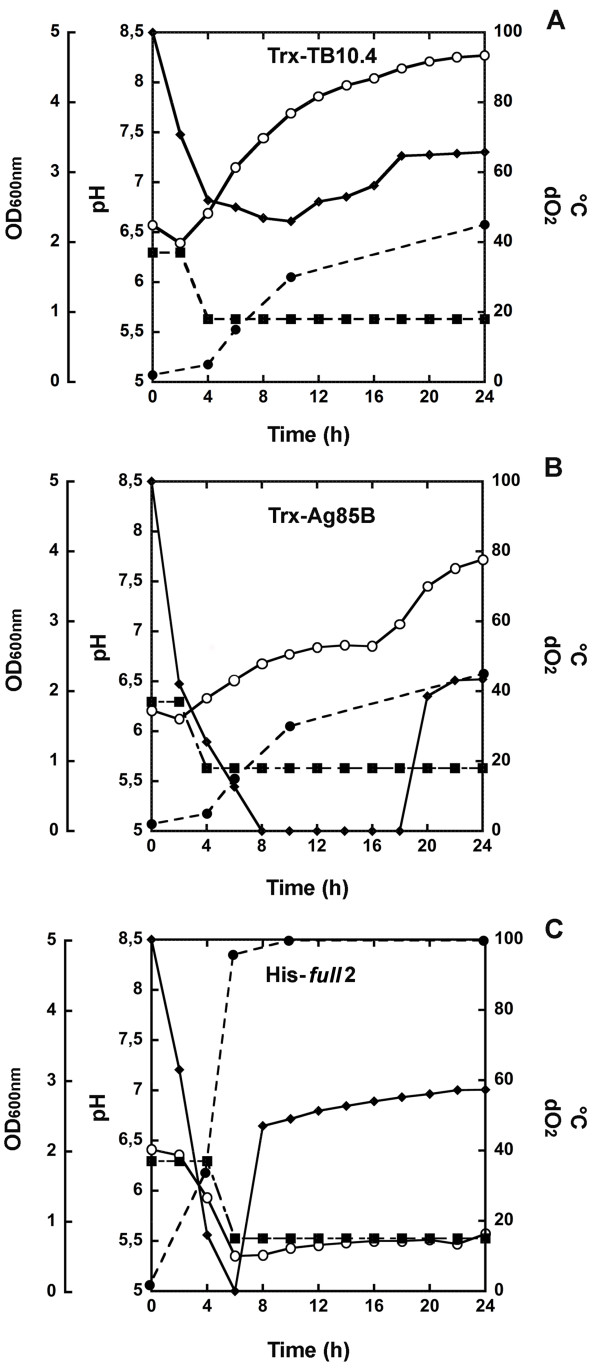
**Time course of pH (○, solid line), dO**_**2 **_**(♦, solid line) and OD**_**600nm **_**(●, dashed line) in 3 L batch cultivation trials of recombinant *E. coli *BL21(DE3) cells producing *M. tuberculosis *immunogenic proteins. A)***E. coli* BL21(DE3) cells containing pET32b-Trx-TB10.4 plasmid and grown in LB medium. **B)***E. coli* BL21(DE3) cells containing pET32b-Trx-Ag85B plasmid and grown in SB medium. **C)***E. coli* BL21(DE3) cells containing pColdI-His-*full*2 and growing in SB/NaCl medium. Temperature (■, dashed line) was kept constant at 37°C before IPTG addition and then reduced to 18°C for Trx-TB10.4 and Trx-Ag85B production, and at 15°C for His-*full*2 production, until harvest. The induction of protein expression was done at OD_600nm_ = 0.8 with 0.1 mM IPTG for Trx-TB10.4, at OD_600nm_ = 2 with 0.1 mM IPTG for Trx-Ag85B, and at OD_600nm_ = 2 with 0.1 mM IPTG for His-*full*2. Biomass (wet weight) collected at the harvest time was 8.5 g/L for Trx-TB10.4, 19.5 g/L for Trx-Ag85B and 6.5 g/L for His-*full*2.

After optimization at the shaken flask-scale, processes and production media were scaled up in 3 L bioreactor. As shown by the parameters controlled on line (dO_2_ and pH) and by densitometric analysis of cell growth during batch cultivation (Figure 5), the growth of recombinant *E. coli* BL21(DE3) cells holding the pColdI-His-*full*2 was faster than those transformed by pET32b-Trx-TB10.4 and pET32b-Trx-Ag85B, even if a lower temperature was adopted after induction of protein expression (15°C for His-*full*2 production vs. 18°C for Trx-TB10.4 and Trx-Ag85B). In 6 hours from *inoculum*, maximum biomass was achieved and dO_2_ was completely depleted in cells producing His-*full*2. In Trx-Ag85B production, oxygen depletion occurred after 8 hours from *inoculum* and the phase of oxygen limitation lasted for further ten hours. *E. coli* Trx-TB10.4-producing cells showed a similar time course of oxygen consumption, but the levels of dO_2_ never decreased below 50% of saturation, indicating a minor respiratory activity; growth of the culture was slower and reached a lower level of biomass production (in wet weight: 19.5 and 8.5 g/L were recovered after 24 hours from cells producing Trx-Ag85B or Trx-TB10.4, respectively, see Table 2). For both Trx-Ag85B or Trx-TB10.4 producing cells, after an initial slight decrease, pH tended to alkalinization in the following fermentation phase. In cells producing His-*full*2, a marked acidification of medium and a rapid re-increase in oxygen level after the initial sharp reduction occurred, probably reflecting a stress status arresting cell proliferation after IPTG addition and growth temperature reduction. Notwithstanding the higher OD_600nm_ of pColdI-His-*full*2 containing cells, final biomass was only 6.5 g/L in wet weight (Table 2).

Table 2 shows that recombinant *E. coli* cells produced more biomass in the stirred bioreactors than in the shaken flasks, due to the better oxygen distribution in the larger scale system which sustains higher density cultivation. Application of the same protocols of metal-chelating chromatography described above to purify Trx-Ag85B, Trx-TB10.4 or His-*full*2 gave a significantly increased purification yield from biomass generated in bioreactor than from shaken flasks: 9.4 mg pure Trx-TB10.4, 1.5 mg pure Trx-Ag85B and 0.38 mg pure His-*full*2 per gram of biomass were achieved at bioreactor scale compared to figures of 2.5 mg Trx-TB10.4, 0.85 mg Trx-Ag85B and 0.076 mg His-*full*2 per gram of biomass obtained at flask-scale. These data indicate a beneficial effect of scaling up on both the volumetric productivity (mg of protein per liter of cultivation broth) and the specific productivity (mg of protein per gram of biomass). In the case of His-*full*2, protein purification was feasible only at the bioreactor scale, due to low specific productivity of cells grown in flasks: the soluble form of His-*full*2 increased from 5 to *ca*. 15% of the total expressed chimeric protein shifting from flasks to bioreactor. In the case of Trx-TB10.4 and Trx-Ag85B, the step of EK digestion and mature protein recovery was not affected by the scaling up, being of about one third for TB10.4 and 50% for Ag85B independently if biomass was generated in flasks or in bioreactors. Identity of the fused and mature antigens was confirmed by MS analyses (spectra overlap with those reported in Figure 2A and C, Figure 3A and B, and Figure 4).

### Biochemical and immunological characterization of the protein antigens

**Figure 6:**
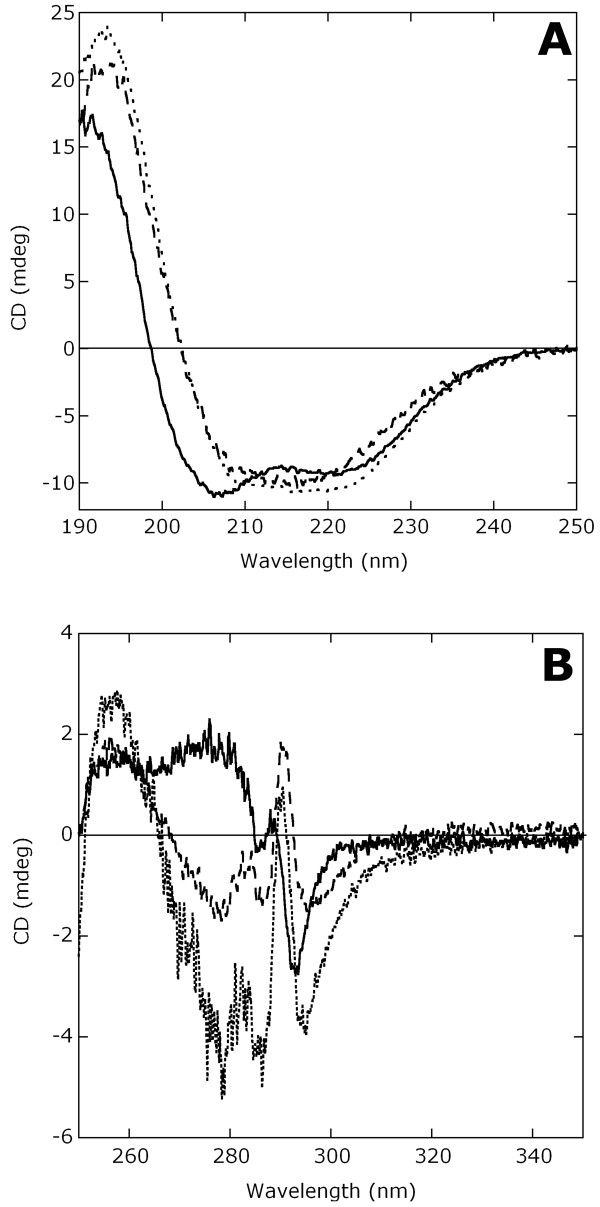
**CD spectra of TB10.4 (continuous line), Ag85B (dashed line) and His-*full*2 (dotted line). A)** Far-UV CD spectra (protein concentration: 0.1 mg/mL). **B)** Near-UV CD spectra (protein concentrations: 0.45, 0.35 and 0.85 mg/mL for TB10.4, Ag85B and His-*full*2, respectively). All spectra were collected in 10 mM ammonium acetate, pH 7, at 15°C.

The quality of the recombinant antigenic proteins was assessed in terms of purity by SDS-PAGE (Additional file 2: Figure S7) and MS analyses (see above) and in terms of protein conformation by circular dichroism (CD) spectroscopy. The secondary structure of pure Ag85B (as determined by the far-UV CD spectra reported in Figure 6A) is composed of both α-helices and β-sheets (*ca*. 26 and 23% as estimated by k2D2 software vs. 37 and 20% from the crystal structure, pdb code 1F0N), while for TB10.4 the signal for α-helices only is apparent, in good agreement with the known 3D structure (55% α-helix content, pdb code 1F0N) and previous analyses [12]. Similarly, the signal for the tertiary structure significantly differs for the two *M. tuberculosis* antigens, see Figure 6B. The near- and far-UV spectra of His-*full*2 resemble those of Ag85B, according to the fact that Ag85B molecular mass is 3-fold higher than that of TB10.4 (Table 1). All together, spectral analyses indicate the acquisition of a well defined protein conformation for all the recombinant soluble antigenic proteins.

**Figure 7:**
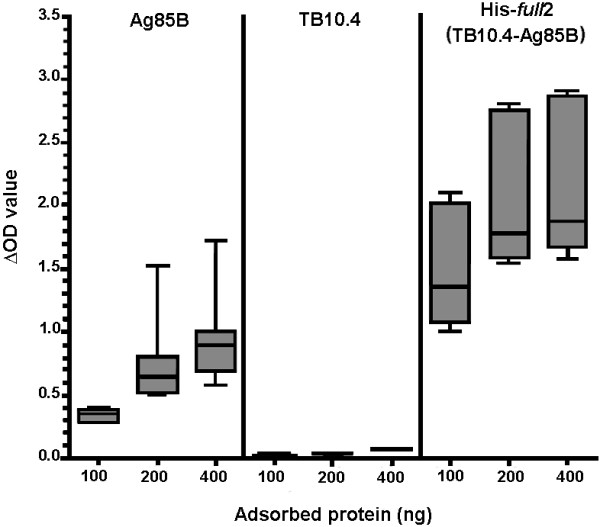
**Analysis of the antibody reactivity to the expressed protein antigens.** Ag85B, TB10.4 and His-*full*2 proteins were adsorbed at different concentrations on ELISA plate (as indicated on abscissa) and tested with active-TB sera at 1:100 dilution. The reactivity is reported as difference of the OD (ΔOD) observed for the sera and in absence of sera as median, 25–75 percentile range (box), and minimum and maximum (line) observed for the 8 tested active-TB patients.

Immunological activity of the recombinant proteins was then assessed by the capability of the proteins to be recognised as antigen by sera (ELISA) or T cells (ELISPOT) of active-TB patients. In a first set of ELISA tests, variable concentrations of the antigens (100, 200 and 400 ng) were adsorbed and tested with TB sera at 1:100 dilution. For the Ag85B and the His-*full*2 proteins, a dose dependent reactivity of TB sera vs. the concentration of the antigen adsorbed on the ELISA plate was observed (Figure 7). On the contrary, almost no reactivity was observed for the TB10.4 protein, in agreement with previous studies [6-8]. Furthermore, in order to assess the specificity of the observed reactivity against the *M. tuberculosis* recombinant Ag85B and His-*full*2 protein antigens, antibody titre was determined for each control and active-TB serum by end final point dilution test. As shown in Additional file 3: Figure S8, TB sera present a significant higher antibody titre (median 600) than controls (median 100, p < 0.001) for the Ag85B protein antigen. Similar results were obtained with the chimeric His-*full*2 protein (TB patients median titre 1200, controls median titre 200, p < 0.001). These data were further confirmed by analysing the single control and active-TB sera reactivity at 1:200 dilution, as reported in Additional file 3: Figure S9.

**Figure 8:**
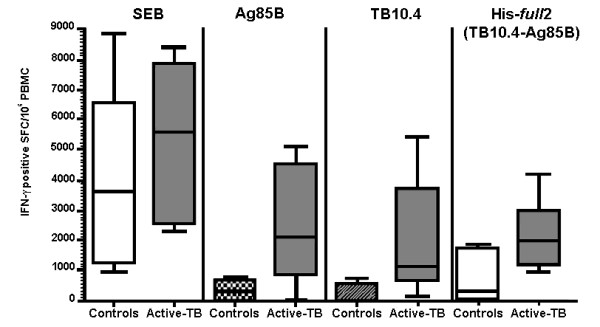
**Analysis of the T-cell reactivity against Ag85B, TB10.4 and His-*full*2 proteins and positive control superantigen (SEB).** Control (white boxes) and active-TB (grey boxes) PBMCs were tested in ELISPOT and the IFN-γ positive effector T-cells enumerated and referred to 1×10^6^ PBMCs (ordinate). The number of spots observed in absence of antigen was subtracted. Data are reported as median, 25–75 percentile range (box), and minimum and maximum (line) observed in subject groups of the study population presenting an immunological valid test (active-TB n = 10; healthy controls n = 6).

Figure 8 shows the results of the T-cell reactivity observed in active TB-patients and healthy controls. Control and active-TB subjects presented a similar T-cell response capability for the *Staphylococcus aureus* Enterotoxin B (SEB) superantigen used as positive control. For all of the *M. tuberculosis* recombinant proteins tested, a significant higher number of IFN-γ producing effector T-cells were observed in active-TB patients compared to MTB-unexposed controls -Ag85B: controls 312 ± 363 (mean ± std) spots/million peripheral blood mononuclear cells (PBMCs), active-TB 2361 ± 1863; p < 0.0001; TB10.4: controls 184 ± 209, active-TB 2009 ± 1901; p < 0.0001; His-*full*2: controls 701 ± 859, active-TB 22183 ± 1173; p = 0.0381 - confirming that the cell mediated immune response is effectively triggered by the antigens produced during this study.

## Discussion

A number of interlinking cells and mechanisms are involved in the immune response following *M. tuberculosis* infection. Delivering effective hits to infected target cells represents the way to push the host response to control infection and, possibly, to eradicate bacteria. At present, the development of new TB vaccines follows two main approaches: a) replacing BCG by either improved recombinant BCG or by genetically attenuated *M. tuberculosis*; b) developing subunit vaccines mostly based on recombinant proteins. In past years, significant progress has been made in producing recombinant antigenic proteins, mostly fusion proteins that combine immunodominant antigens of *M. tuberculosis, e.g.*, the Hybrid 1 fusion protein which consists of Ag85B fused to ESAT6, or Hyvac 4 consisting of Ag85B fused to the antigen TB10.4 [16]. Our aim is to produce well-known *M. tuberculosis* antigens with a native-like structure in order to preserve conformational epitopes in addition to the sequential ones. The importance of native-like structure has been made apparent by the structural vaccinology approach, in which the three-dimensional structural information is used to design novel and improved vaccine antigens. As stated by [17]: *“We now routinely determine structures of vaccine antigens as a central task in vaccine optimization. The essential insights that antibody epitopes are conformational, extended protein surfaces and that short, floppy peptides rarely elicit protective antibodies are essential for selecting vaccine research strategies that have a high probability of success”.*

In the present study, solubility enhancement by N-terminal thioredoxin fusion and/or by optimization of fermentation conditions was investigated for the recombinant antigens Ag85B, TB10.4 and *full* (TB10.4-Ag85B). Protein over-expression in *E. coli* frequently leads to the formation of IBs; obtaining proteins from the IBs has several drawbacks. Despite these disadvantages, the recombinant *M. tuberculosis* antigens Ag85B [18], TB10.4 [19], and Ag85B-TB10.4 [6] have been previously purified from IBs and formulated into subunit vaccines although their immunogenicity was not the same as for the corresponding native antigens or soluble counterparts. A comparison was recently carried out for TB10.4 and TB10.4-Ag85B proteins [12] and, interestingly, re-solubilized TB10.4-Ag85B purified from IBs did not recover the conformation adopted by its counterpart purified from the soluble fraction, but it seemed to adopt an intermediate conformation. In order to preserve native conformation, we produced the three antigens as soluble proteins. The overall yields hereby achieved favourably compare with the values reported in literature: 5 and 1 mg of mature Ag85B and TB10.4-Ag85B per liter of cultivation broth were previously produced in soluble form [12] vs. 16 and 2.5 mg/L obtained in our conditions (see Table 2); to our knowledge, literature data concerning the production of mature TB10.4 as soluble protein (vs. 25 mg/L with our procedure) were not reported.

Circular dichroism spectroscopy was used to study secondary and tertiary protein structure. Soluble Ag85B and His-*full*2 proteins have similar CD spectra. They closely resemble those obtained previously for soluble antigens and distinguished from TB10.4-Ag85B purified from IBs [12]. Our procedure based on the isolation of the proteins from the soluble fraction avoided the folding drawbacks encountered when the TB10.4-Ag85B chimeric protein was re-solubilized from IBs [12].

The produced recombinant proteins presented an appropriate immunogenicity as for the capability to be recognised either by antibodies and T-cells of active-TB patients. Interestingly, when TB10.4 was fused to Ag85B, it presented a stronger immune-reactivity respect the sum of the single proteins (see end final point dilution test in Additional file 3), likely due to a better accessibility of the antigen B-cell epitope recognised by the TB-patient antibody. A similar result was reported earlier [6] also showing that TB10.4 distinguished from ESAT-6, which appeared to be subdominant when fused to Ag85B. Although less apparent, a similar result was also achieved for the T-cell reactivity test (Figure 8). All together, the fusion of TB10.4 with Ag85B did not decrease the immunogenicity of either TB10.4 or Ag85B and has a beneficial effect on the immunogenicity of TB10.4.

## Conclusions

In conclusion, the main purpose of this study to purify large quantities of soluble antigens effective in immunological response against *M. tuberculosis* was reached by the rational design of the expression constructs and the optimization of fermentation conditions for *E. coli* recombinant strains. The set up of a controlled, scalable, robust and economically feasible production process (current cost of antigenic protein production at the lab scale is 50–100 €/mg, whereas Ag85B is commercially available at ≥ 1800 €/mg) is important for supporting research and development of novel vaccine candidates to prevent and/or treat tuberculosis.

## Methods

### Design, synthesis and cloning of cDNA coding for antigenic proteins

Synthetic cDNAs coding for TB10.4 and Ag85B antigenic proteins were designed *in silico* by back translation of the amino acid sequences reported in the database (TB10.4: GenBank Accession no. CAA17363.1; Ag85B: UniProtKB/Swiss-Prot Accession no. P0C5B9.1). A third cDNA, in which the nucleotide sequence coding for TB10.4 is upstream to the sequence coding for Ag85B (*full* cDNA) was also designed. All synthetic cDNAs were produced by Eurofins Medigenomix GmbH (Ebersberg, Germany), after optimization of the nucleotidic sequences for expression in *E. coli* (see Additional file 2: Figure S1). The three synthetic genes were cloned into pET32b using *Bam*HI and *Eco*RI restriction sites. By this cloning strategy, the genes were fused with thioredoxin (Trx) encoding gene and tagged by an His_6_-tag. Two additional constructs of the *full* cDNA were also produced. To prepare His-*full*1 form, the *full* cDNA was extracted from pET32b plasmid using *Kpn*I and *Eco*RI restriction sites and cloned into pColdI plasmid (TaKaRa Bio Inc., Otsu, Japan). For the production of His-*full*2 form, site-directed mutagenesis was carried out on the *full* cDNA using the QuikChange II Site-Directed Mutagenesis Kit (Agilent Technologies, Santa Clara, CA, USA), to insert a *Nde*I restriction site at the 5′ of the *full* cDNA, including the triplet coding for the initial methionine. The following mutagenesis primers were used: 5′GCCATGAGCTCGGATC*AT*ATGAGCCAGATCATG3′; 5′CATGATCTGGCTCAT*AT*GATCCGAGCTCATGGC3′ (mutated bases are in italics). The presence of the desired mutation was confirmed by automated DNA sequencing. The mutated cDNA was then cloned into pColdI plasmid using *Nde*I and *Eco*RI restriction sites. The gene sequences are reported in Figure 1.

### Strain and growth conditions

For protein expression, all plasmids were transferred to the *E. coli* hosts BL21(DE3) and BL21(DE3)pLysS. The following media were used: M9/glucose (6.78 g/L Na_2_HPO_4_, 3 g/L KH_2_PO_4_, 1 g/L NH_4_Cl, 0.5 g/L NaCl, 0.1 mM CaCl_2_, 2 mM MgSO_4_, 10 g/L glucose); Luria-Bertani (LB, 10 g/L bacto-tryptone, 5 g/L yeast extract, 5 g/L NaCl); Terrific Broth (TB, 12 g/L bacto-tryptone, 24 g/L yeast extract, 8 mL/L glycerol, 2.2 g/L KH_2_PO_4_, 9.4 g/L K_2_HPO_4_); Terrific Broth/NaCl (TB, 12 g/L bacto-tryptone, 24 g/L yeast extract, 8 mL/L glycerol, 2.2 g/L KH_2_PO_4_, 9.4 g/L K_2_HPO_4_, 5 g/L NaCl); Super Broth (SB, 32 g/L bacto-tryptone, 20 g/L yeast extract, 5 g/L NaCl); Super Broth/NaCl (SB/NaCl, 32 g/L bacto-tryptone, 20 g/L yeast extract, 10 g/L NaCl) [15,20]. Starter cultures were prepared from a single colony of *E. coli* carrying the recombinant plasmid, employing the same medium used for protein expression containing ampicillin (100 μg/mL, final concentration) and, in the case of BL21(DE3)pLysS strain, also chloramphenicol (34 μg/mL, final concentration). Cultures were grown overnight under vigorous shaking at 37°C. For chaperone co-expression, BL21(DE3) *E. coli* cells were co-transformed with pKJE7 (coding for AraB, DnaK, DnaJ and GrpE) or with pG-Tf2 (coding for GroES, GroEL and TiG) plasmids (Chaperone Plasmid Set, TaKaRa Bio Inc., Otsu, Japan), adding chloramphenicol (30 μg/mL, final concentration) to the growth medium. Expression of the chaperone proteins was induced by adding 1 mg/mL L-arabinose (for pKJE7) or with 2 ng/mL tetracyclin (for pG-Tf2), according to the manufacturer’s instructions. Protein production trials were carried out in 500 mL or 2 L baffled Erlenmeyer flasks containing 80 mL or 500 mL of each medium inoculated with the starter cultures (initial OD_600nm_ = 0.1). Cells were grown at 37°C with shaking (150 rpm) until protein expression was induced by adding IPTG: cells were then grown at 15 or 18°C until harvesting. Collected cells were washed with STE buffer and stored at -20°C.

### 3 L bioreactor cultivations

SB for *E. coli* BL21(DE3) cells containing the pET32b-Trx-Ag85B plasmid, LB for those transformed with pET32b-Trx-TB10.4 plasmid and SB/NaCl for those transformed with pColdI-His-*full*2 plasmid were used as production media in 2 L working volume P-100 Applikon 3 L glass reactor (height 25 cm, diameter 13 cm) equipped with a AD1030 Biocontroller and AD1032 motor [20,21]. Cultivations in bioreactor were conducted in batch-mode at 37°C, 500 rpm stirring (corresponding to 1.17 m/s of tip speed) and 2 L/min aeration rate. Foam production was controlled by the addition of Hodag antifoam through an antifoam sensor. Starter cultures were grown overnight in SB, LB or SB/NaCl medium and diluted up to an initial OD_600nm_ of 0.1. For pET32b-Trx-Ag85B and pET32b-Trx-TB10.4 recombinant cells, after 2–4 hours of growth (corresponding to an OD_600nm_ of 2 for the previous and of 0.8 for the latter), 0.1 mM IPTG was added and the temperature was decreased to 18°C. pColdI-His-*full*2 recombinant cells grown for 4–6 hours (corresponding to an OD_600nm_ of 2) were added of 0.1 mM IPTG and 25 g/L NaCl and the temperature was decreased to 15°C. After 16 hours, cells were harvested by centrifugation, washed with STE buffer and stored at -20°C.

### Crude extract preparation and purification of fusion proteins

Cell paste was re-suspended in phosphate buffer saline (PBS) (8.1 mM Na_2_HPO_4_, 1.76 mM KH_2_PO_4_, 0.137 M NaCl, 2.68 mM KCl), pH 7.0, 1 mM phenylmethanesulphonylfluride (PMSF), 10 μg/mL deoxyribonuclease. Cells were disrupted by sonication (5 cycles of 30 s each, in ice, with 30-s interval). The insoluble fraction was removed by centrifugation at 39000 × *g* for 1 hour at 4°C. The crude extract (added of 0.9 M NaCl) was loaded on a HiTrap Chelating column (GE Healthcare, Piscataway, NJ, USA), pre-loaded with Ni^2+^ and equilibrated in PBS, 0.9 M NaCl, pH 7.0. Elution of the fusion protein was performed increasing the amount of PBS buffer containing 0.5 M imidazole, pH 7.0 [22]. The pure proteins were then equilibrated in 20 mM TrisHCl, 50 mM NaCl, pH 7.4 by size-exclusion chromatography on a PD10 desalting column or by extensive dialysis.

### Enterokinase cleavage of the fusion proteins

Enterokinases (EK, Recombinant Enterokinase, and Tag · off High Activity Recombinant Enterokinase) and Recombinant Enterokinase Capture Agarose were purchased from Novagen. One EK unit is defined as the amount of enzyme that cleaves 50 μg of a control protein (supplied by the producer) in 16 hours at room temperature and in 20 mM TrisHCl, 50 mM NaCl, 2 mM CaCl_2_, pH 7.4.

### SDS-PAGE electrophoresis and Western blot analysis

Proteins from total cell extracts or from both soluble and insoluble cell fractions were separated by SDS-PAGE: gels were stained for proteins with Coomassie Blue R-250. For total cell extracts and for insoluble fractions after cell disruption, cell pellets were directly resuspended in an appropriate volume of Laemmli sample buffer. For Western blot analysis, upon separation by SDS-PAGE, proteins were transferred electrophoretically onto a nitrocellulose membrane. The fusion proteins were detected using anti-His-tag mouse monoclonal antibody (His-probe, Santa Cruz Biotechnology, Santa Cruz, CA, USA), rabbit anti-TB10.4 polyclonal antibodies (Antibodies-online GmbH, Aachen, Germany), or rabbit anti-Ag85B polyclonal antibody (DIATHEVA, Fano, Italy), in combination with the appropriate secondary antibody: goat anti-mouse (Santa Cruz Biotechnology, Santa Cruz, CA, USA), donkey anti-mouse and donkey anti-rabbit (Jackson ImmunoResearch Laboratories Inc., West Grove, PA, USA) IgG HRP-conjugated antibody. The immunorecognition was detected by a chemioluminescence method (ECL Plus Western Blotting Detection System, GE Healthcare, Piscataway, NJ, USA). The amount of protein was estimated by evaluating the intensity of the signal using the public domain, Java-based image processing program ImageJ (National Institutes of Health, http://rsb.info.nih.gov/ij/). His-tagged D-amino acid oxidase [23] was used as standard.

### Spectral measurements

Extinction coefficients of native proteins were determined using a Jasco V-560 spectrophotometer by complete denaturation of appropriate dilutions of protein samples in 6 M urea assuming as extinction coefficients of the fully denatured proteins those calculated by ExPASy Bioinformatic Resource Portal (http://www.expasy.org/) using ProtParam tool [24].

Circular dicroism (CD) spectra were recorded on a J-815 Jasco spectropolarimeter [24]: cell path-lenth was 1 cm for measurements above 250 nm (0.4 mg protein/mL) and 0.1 cm for measurements in the 190–250 nm range (0.1 mg protein/mL). Secondary structure fractions were calculated from deconvolution of the CD spectra with the program K2D2 (http://www.ogic.ca/projects/k2d2/) [25].

Temperature ramp experiments were carried out by using the same instrumentation equipped with a software-driven Peltier-based temperature controller (temperature gradient: 0.5°C/min). All spectral experiments were carried out in 20 mM MOPS, 0.4 M NaCl, pH 7.0 or in 10 mM ammonium acetate, pH 7.0 at 15°C.

### Mass spectrometry analyses

Intact MS experiments were in general carried out on a LTQ-MS (Thermo Electron, San Jose, CA, USA) with an ESI source. The protein samples were prepared in 10 mM ammonium bicarbonate (pH 7.4), 0.1% acetonitrile/trifluroacetic acid (50/50) to a final concentration of 0.3 mg/mL. Full scan intact MS experiments were carried out under the following instrumental conditions: positive ion mode, mass range 700–2000 m/z, source voltage 4.5 kV, capillary voltage 35 V, sheat gas 15, auxiliary gas 2, capillary temperature 220°C, tube lens voltage 140 V. Five spectra were acquired for each data and each spectrum was the composite of two averaged scans. Data processing was performed using Bioworks Browser (Thermo Electron, revision 3.1). Theoretical average masses were calculated by Ion Source Bioinformatic Resource Portal (http://www.ionsource.com/) using Peptide Mass Calculator tool. Identification of truncated forms of the proteins was carried out by MS-non specific database search program Protein Prospector v 5.10.4 (http://prospector.ucsf.edu).

The His-*full2* protein was dissolved in acetonitrile and analyzed using a MALDI-TOF/TOF Ultraflex III spectrometer (Bruker Daltonics, Bremen, Germany) in linear positive mode. A solution of sinapinic acid (SA) 20 mg/mL solved in a 70/30 acetonitrile/trifluoroacetic acid (TFA) 0.1% solution, was used as matrix. The spectra were acquired in a mass range of 6000–52000 m/z and processed using Flex Analysis software 3.0 (Bruker Daltonics).

### Immunological studies: study population

The study population included 19 subjects. Twelve patients with newly diagnosed, untreated active pulmonary TB and 7 healthy individuals without any history of TB exposure (hereafter indicated as TB unexposed controls). Study subjects were recruited at the Department of Infectious Diseases of the Fondazione IRCCS-Policlinico San Matteo of Pavia (Italy), after informed consent. In all cases, the diagnosis of active TB was confirmed by *M. tuberculosis* culture isolation.

Peripheral venous blood was obtained for serum samples from all the participants to the study to perform ELISA assay. Additionally, peripheral blood was collected into tubes containing heparin for ELISPOT assay. Peripheral blood mononuclear cells (PBMC) were isolated by standard density gradient centrifugation using Lymphoprep (Axis-Shield, Oslo, Norway). Isolated PBMC were cryopreserved in freezing medium: 10% v/v DMSO (Sigma-Aldrich, St. Louis, MO, USA), 25% v/v human albumin (Grifolds Biologicals, Los Angeles, CA, USA) and 65% v/v RPMI 1640 supplemented with 2 mM L-glutamine, 100 U/mL penicillin and 100 μg/mL streptomycin (all from Euroclone, Milan, Italy) and kept in liquid nitrogen until ELISPOT analyses.

### ELISA assay

ELISA assay was performed with the method of [26] with minor modifications. Microplates 96-wells high-binding capacity flat-bottom (Greiner-bio-one GmbH, Frickenhausen Germany) were coated with different concentrations (400, 200, 100 ng/well) of Ag85B, TB10.4, His-*full*2 in 50 mM bicarbonate buffer (pH 9.6) and incubated at room temperature overnight. Plates were washed three times with 0.05% (v/v) Tween-20 in phosphate buffered saline (PBS-T, Sigma-Aldrich, St. Louis, MO, USA) and incubated with 200 μL/well of blocking buffer (PBS with 1% BSA) at room temperature for at least 1 hour and then washed three times with PBS-T. Serum samples were diluted with dilution buffer (PBS-T with 1% BSA) at scalar dilution of 1:50 to 1:800. One-hundred microliter of the diluted sera was added to each well and incubated for 1 hour at 37°C. Plates were then washed four times with PBS-T. Anti-human specific IgG antibody labeled with horseradish peroxidase (MP Biomedicals, LLC Santa Ana, CA, USA) was added to each well and incubated at 37°C for 1 hour. Plates were again washed four times with PBS-T and 100 μL of o-phenyl-diamine/H_2_O_2_ substrate (Sigma-Aldrich, St. Louis, MO, USA) was added for 30 minutes. The reaction was stopped by adding 50 μL of 0.5 M H_2_SO_4_. The absorbance at 492 nm was measured with an ELISA plate spectrophotometer (Titertek Plus MS212 ICN, Eschwege, Germany).

### Evaluation of peptide specific single T-cell IFN-γ release

The enumeration of peptide specific T-cell producing IFN-*γ* was performed at the single cell level by using ELISPOT assay, as previously described, with minor modifications [27].

In detail, PBMC were thawed, washed and re-suspended in the culture medium: RPMI 1640 medium supplemented with 2 mM L-glutamine, 100 U/mL penicillin, 100 μg/mL streptomycin and 10% heat-inactivated fetal calf serum (Euroclone, Milan, Italy). Cells were kept overnight at 37°C in a humidified 5% CO_2_ atmosphere. Cells were then transferred to a 24-well flat-bottom plate (5 × 10^5^ cells/mL per well), stimulated with 1 μg/mL of each protein (one protein per well) or culture medium only or phytohemagglutinin (PHA) (5 μg/mL; Sigma-Aldrich, St. Louis, MO, USA), and cultured at 37°C in a humidified 5% CO_2_ atmosphere for 10 days. On days 3 and 7, half of the supernatant from each well was removed and replaced with wet culture medium supplemented with 20 IU/mL recombinant human IL-2 (Peprotech, London, UK). After 10 days, cells from each well were harvested, washed three times with culture medium and re-suspended at a concentration of 4 × 10^5^ cells/mL before their use in ELISPOT assay. Un-stimulated PBMC were included as negative controls.

In ELISPOT assay, multiScreen-IP 96-well plates (Merck Millipore, Darmstadt, Germany) were coated with IFN-γ capture antibody and incubated overnight at 4°C. Plates were then washed 5 times with PBS and blocked with culture medium for 2 hours at room temperature. Antigen-stimulated cells for 10 days were added in duplicate (4 × 10^4^ cells/well) and re-stimulated with each protein used for stimulation during the 10-day period. Cells stimulated with PHA during the 10-day period were re-stimulated with *Staphylococcus aureus* Enterotoxin B (SEB) superantigen (2 μg/mL; Sigma-Aldrich, St. Louis, MO, USA). After incubation at 37°C in a humidified 5% CO_2_ atmosphere for 24 hours, plates were washed five times with PBS-T (Sigma-Aldrich, St. Louis, MO, USA). Biotinylated detection antibody for IFN-γ was added and incubated overnight at 4°C. After five washes with PBS-T, streptavidin-alkaline phosphatase conjugate was added and plates were incubated at 37°C, 5% CO_2_ atmosphere for 1 hour. Plates were then washed, 5-bromo-4-chloro-3-indolyl-phosphate/nitro blue tetrazolium (BCIP/NBT) was added and incubated at room temperature for 20 minutes. Wells were then washed several times under running water and air-dried. Spot-forming cells (SFCs) were counted in duplicate wells using an automated ELISPOT reader system (Autoimmun Diagnostika GmbH, Strassberg, Germany). The mean value of the SFCs was calculated, and the value of the control subtracted to the SFCs of the stimuli: values were referred to million PBMC.

### Statistical analysis

Data are expressed using mean and standard deviation of the mean or median and percentiles as appropriate. Comparison between groups have been made using Mann–Whitney and χ^2^ tests. A *p* value below 0.05 was considered significant. All tests were performed using the GraphPad Prism 4.0 (Graphpad software, San Diego, CA, USA) software package.

## Competing interests

The authors declare that they have no competing interests.

## Author’s contributions

LuP and MC cloned, expressed and purified the chimeric proteins. CT and MT developed the analytical methods for protein identity and purity. EB and FlM carried out experiments at fermenter scale. FrM and MA performed the immunological assays and analyzed the data. FlM and LoP co-wrote the paper. LoP supervised the protein biochemistry experiments and conceived the project. All authors have read and approved the final manuscript.

## Supplementary Material

Additional file 1 Nucleotide and amino acid sequences of antigenic proteins. Click here for file

Additional file 2 Expression and purification of antigenic proteins. Click here for file

Additional file 3 Immunological assays. Click here for file
