# Supplementary material for: Optimizing Escherichia coli as a protein expression platform to produce Mycobacterium tuberculosis immunogenic proteins
Source: Microb Cell Fact. 2013 Nov 19;12:115. doi: 10.1186/1475-2859-12-115 (PMC4225511; doi:10.1186/1475-2859-12-115)
Supplement: Additional file 1 — Nucleotide and amino acid sequences of antigenic proteins. [file 1475-2859-12-115-S1.pdf]

Additional File 1. Nucleotide and amino acid sequences.

Figure S1

TB10.4 gene:

```
      BamHI
      GGATCCAATGAGCCAGATCATGTATAACTATCCGGCAATGCTGGGTCATGCCGGTGATATGGCAGGTTATGCAGGCACCC
   1  ---------+---------+---------+---------+---------+---------+---------+---------+
      CCTAGGTTACTCGGTCTAGTACATATTGATAGGCCGTTACGACCCAGTACGGCCACTATACCGTCCAATACGTCCGTGGG
             M__S__Q__I__M__Y__N__Y__P__A__M__L__G__H__A__G__D__M__A__G__Y__A__G__T__L

      TGCAGAGCCTGGGTGCAGAAATTGCAGTTGAACAGGCAGCACTGCAGAGCGCATGGCAGGGTGATACCGGTATTACCTAT
  81  ---------+---------+---------+---------+---------+---------+---------+---------+
      ACGTCTCGGACCCACGTCTTTAACGTCAACTTGTCCGTCGTGACGTCTCGCGTACCGTCCCACTATGGCCATAATGGATA
      __Q__S__L__G__A__E__I__A__V__E__Q__A__A__L__Q__S__A__W__Q__G__D__T__G__I__T__Y__

      CAGGCATGGCAGGCACAGTGGAATCAGGCAATGGAAGATCTGGTTCGTGCATATCATGCAATGAGCAGCACCCATGAAGC
 161  ---------+---------+---------+---------+---------+---------+---------+---------+
      GTCCGTACCGTCCGTGTCACCTTAGTCCGTTACCTTCTAGACCAAGCACGTATAGTACGTTACTCGTCGTGGGTACTTCG
      Q__A__W__Q__A__Q__W__N__Q__A__M__E__D__L__V__R__A__Y__H__A__M__S__S__T__H__E__A_

                                                                   EcoRI
      AAATACCATGGCAATGATGGCACGTGATACCGCTGAAGCAGCAAAATGGGGTGGTTAAGAATTC
 241  -------+---------+---------+---------+---------+---------+------  304
      TTTATGGTACCGTTACTACCGTGCACTATGGCGACTTCGTCGTTTTACCCCACCAATTCTTAAG
      _N__T__M__A__M__M__A__R__D__T__A__E__A__A__K__W__G__G__*__
```

Ag85B gene:

```
      BamHI
      GGATCCATTTAGCCGTCCGGGTCTGCCGGTTGAATATCTGCAGGTTCCGAGCCCGAGCATGGGTCGTGATATTAAAGTTC
   1  ---------+---------+---------+---------+---------+---------+---------+---------+
      CCTAGGTAAATCGGCAGGCCCAGACGGCCAACTTATAGACGTCCAAGGCTCGGGCTCGTACCCAGCACTATAATTTCAAG
             F__S__R__P__G__L__P__V__E__Y__L__Q__V__P__S__P__S__M__G__R__D__I__K__V__Q

      AGTTTCAGAGCGGTGGCAATAATAGTCCGGCAGTTTATCTGCTGGATGGTCTGCGTGCACAGGATGATTATAATGGTTGG
  81  ---------+---------+---------+---------+---------+---------+---------+---------+
      TCAAAGTCTCGCCACCGTTATTATCAGGCCGTCAAATAGACGACCTACCAGACGCACGTGTCCTACTAATATTACCAACC
      __F__Q__S__G__G__N__N__S__P__A__V__Y__L__L__D__G__L__R__A__Q__D__D__Y__N__G__W__

      GATATTAACACCCCTGCCTTTGAATGGTATTACCAGAGCGGTCTGAGCATTGTTATGCCGGTTGGTGGTCAGAGCAGCTT
 161  ---------+---------+---------+---------+---------+---------+---------+---------+
      CTATAATTGTGGGGACGGAAACTTACCATAATGGTCTCGCCAGACTCGTAACAATACGGCCAACCACCAGTCTCGTCGAA
      D__I__N__T__P__A__F__E__W__Y__Y__Q__S__G__L__S__I__V__M__P__V__G__G__Q__S__S__F_

      TTATAGCGATTGGTATAGTCCGGCATGTGGTAAAGCAGGTTGTCAGACCTATAAATGGGAAACCTTTCTGACCAGCGAAC
 241  ---------+---------+---------+---------+---------+---------+---------+---------+
      AATATCGCTAACCATATCAGGCCGTACACCATTTCGTCCAACAGTCTGGATATTTACCCTTTGGAAAGACTGGTCGCTTG
      _Y__S__D__W__Y__S__P__A__C__G__K__A__G__C__Q__T__Y__K__W__E__T__F__L__T__S__E__L

      TGCCGCAGTGGCTGAGCGCAAATCGTGCAGTTAAACCGACCGGTAGCGCAGCAATTGGTCTGAGCATGGCAGGTAGCAGC
 321  ---------+---------+---------+---------+---------+---------+---------+---------+
      ACGGCGTCACCGACTCGCGTTTAGCACGTCAATTTGGCTGGCCATCGCGTCGTTAACCAGACTCGTACCGTCCATCGTCG
      __P__Q__W__L__S__A__N__R__A__V__K__P__T__G__S__A__A__I__G__L__S__M__A__G__S__S__

      GCAATGATTCTGGCAGCATATCATCCGCAGCAGTTTATCTATGCAGGTAGCCTGAGCGCACTGCTGGACCCGAGCCAGGG
 401  ---------+---------+---------+---------+---------+---------+---------+---------+
      CGTTACTAAGACCGTCGTATAGTAGGCGTCGTCAAATAGATACGTCCATCGGACTCGCGTGACGACCTGGGCTCGGTCCC
      A__M__I__L__A__A__Y__H__P__Q__Q__F__I__Y__A__G__S__L__S__A__L__L__D__P__S__Q__G_

      TATGGGTCCGAGCCTGATTGGTCTGGCAATGGGTGATGCCGGTGGTTATAAAGCAGCAGATATGTGGGGTCCGAGCAGCG
 481  ---------+---------+---------+---------+---------+---------+---------+---------+
      ATACCCAGGCTCGGACTAACCAGACCGTTACCCACTACGGCCACCAATATTTCGTCGTCTATACACCCCAGGCTCGTCGC
```

```
     _M__G__P__S__L__I__G__L__A__M__G__D__A__G__G__Y__K__A__A__D__M__W__G__P__S__S__D
```

```
     ATCCGGCATGGGAACGTAATGATCCGACCCAGCAGATTCCGAAACTGGTTGCAAATAATACCCGTCTGTGGGTTTATTGT
561  ---------+---------+---------+---------+---------+---------+---------+---------+
     TAGGCCGTACCCTTGCATTACTAGGCTGGGTCGTCTAAGGCTTTGACCAACGTTTATTATGGGCAGACACCCAAATAACA
     __P__A__W__E__R__N__D__P__T__Q__Q__I__P__K__L__V__A__N__N__T__R__L__W__V__Y__C__

     GGTAATGGCACCCCGAATGAACTGGGTGGTGCAAATATTCCGGCAGAATTTCTGGAAAATTTTGTGCGTAGCAGCAACCT
641  ---------+---------+---------+---------+---------+---------+---------+---------+
     CCATTACCGTGGGGCTTACTTGACCCACCACGTTTATAAGGCCGTCTTAAAGACCTTTTAAAACACGCATCGTCGTTGGA
     G__N__G__T__P__N__E__L__G__G__A__N__I__P__A__E__F__L__E__N__F__V__R__S__S__N__L_

     GAAATTCCAGGATGCATATAATGCAGCCGGTGGTCATAATGCCGTTTTTAACTTTCCGCCTAATGGCACCCATAGCTGGG
721  ---------+---------+---------+---------+---------+---------+---------+---------+
     CTTTAAGGTCCTACGTATATTACGTCGGCCACCAGTATTACGGCAAAAATTGAAAGGCGGATTACCGTGGGTATCGACCC
     _K__F__Q__D__A__Y__N__A__A__G__G__H__N__A__V__F__N__F__P__P__N__G__T__H__S__W__E

                                                                         EcoRI
     AATATTGGGGTGCACAGCTGAATGCAATGAAAGGTGATCTGCAGAGCAGCCTGGGTGCAGGTTAAGAATTC
801  ---------+---------+---------+---------+---------+---------+---------+-  871
     TTATAACCCCACGTGTCGACTTACGTTACTTTCCACTAGACGTCTCGTCGGACCCACGTCCAATTCTTAAG
     __Y__W__G__A__Q__L__N__A__M__K__G__D__L__Q__S__S__L__G__A__G__*__
```

"*full*" gene:

```
     BamHI
     GGATCCAATGAGCCAGATCATGTATAACTATCCGGCAATGCTGGGTCATGCCGGTGATATGGCAGGTTATGCAGGCACCC
  1  ---------+---------+---------+---------+---------+---------+---------+---------+
     CCTAGGTTACTCGGTCTAGTACATATTGATAGGCCGTTACGACCCAGTACGGCCACTATACCGTCCAATACGTCCGTGGG
           M__S__Q__I__M__Y__N__Y__P__A__M__L__G__H__A__G__D__M__A__G__Y__A__G__T__L

     TGCAGAGCCTGGGTGCAGAAATTGCAGTTGAACAGGCAGCACTGCAGAGCGCATGGCAGGGTGATACCGGTATTACCTAT
 81  ---------+---------+---------+---------+---------+---------+---------+---------+
     ACGTCTCGGACCCACGTCTTTAACGTCAACTTGTCCGTCGTGACGTCTCGCGTACCGTCCCACTATGGCCATAATGGATA
     __Q__S__L__G__A__E__I__A__V__E__Q__A__A__L__Q__S__A__W__Q__G__D__T__G__I__T__Y__

     CAGGCATGGCAGGCACAGTGGAATCAGGCAATGGAAGATCTGGTTCGTGCATATCATGCAATGAGCAGCACCCATGAAGC
161  ---------+---------+---------+---------+---------+---------+---------+---------+
     GTCCGTACCGTCCGTGTCACCTTAGTCCGTTACCTTCTAGACCAAGCACGTATAGTACGTTACTCGTCGTGGGTACTTCG
     Q__A__W__Q__A__Q__W__N__Q__A__M__E__D__L__V__R__A__Y__H__A__M__S__S__T__H__E__A_

     AAATACCATGGCAATGATGGCACGTGATACCGCTGAAGCAGCAAAATGGGGTGGTTTTAGCCGTCCGGGTCTGCCGGTTG
241  ---------+---------+---------+---------+---------+---------+---------+---------+
     TTTATGGTACCGTTACTACCGTGCACTATGGCGACTTCGTCGTTTTACCCCACCAAAATCGGCAGGCCCAGACGGCCAAC
     _N__T__M__A__M__M__A__R__D__T__A__E__A__A__K__W__G__G__F__S__R__P__G__L__P__V__E

     AATATCTGCAGGTTCCGAGCCCGAGCATGGGTCGTGATATTAAAGTTCAGTTTCAGAGCGGTGGCAATAATAGTCCGGCA
321  ---------+---------+---------+---------+---------+---------+---------+---------+
     TTATAGACGTCCAAGGCTCGGGCTCGTACCCAGCACTATAATTTCAAGTCAAAGTCTCGCCACCGTTATTATCAGGCCGT
     __Y__L__Q__V__P__S__P__S__M__G__R__D__I__K__V__Q__F__Q__S__G__G__N__N__S__P__A__

     GTTTATCTGCTGGATGGTCTGCGTGCACAGGATGATTATAATGGTTGGGATATTAACACCCCTGCCTTTGAATGGTATTA
401  ---------+---------+---------+---------+---------+---------+---------+---------+
     CAAATAGACGACCTACCAGACGCACGTGTCCTACTAATATTACCAACCCTATAATTGTGGGGACGGAAACTTACCATAAT
     V__Y__L__L__D__G__L__R__A__Q__D__D__Y__N__G__W__D__I__N__T__P__A__F__E__W__Y__Y_

     CCAGAGCGGTCTGAGCATTGTTATGCCGGTTGGTGGTCAGAGCAGCTTTTATAGCGATTGGTATAGTCCGGCATGTGGTA
481  ---------+---------+---------+---------+---------+---------+---------+---------+
     GGTCTCGCCAGACTCGTAACAATACGGCCAACCACCAGTCTCGTCGAAAATATCGCTAACCATATCAGGCCGTACACCAT
     _Q__S__G__L__S__I__V__M__P__V__G__G__Q__S__S__F__Y__S__D__W__Y__S__P__A__C__G__K

     AAGCAGGTTGTCAGACCTATAAATGGGAAACCTTTCTGACCAGCGAACTGCCGCAGTGGCTGAGCGCAAATCGTGCAGTT
561  ---------+---------+---------+---------+---------+---------+---------+---------+
     TTCGTCCAACAGTCTGGATATTTACCCTTTGGAAAGACTGGTCGCTTGACGGCGTCACCGACTCGCGTTTAGCACGTCAA
     __A__G__C__Q__T__Y__K__W__E__T__F__L__T__S__E__L__P__Q__W__L__S__A__N__R__A__V__
```

```
        AAACCGACCGGTAGCGCAGCAATTGGTCTGAGCATGGCAGGTAGCAGCGCAATGATTCTGGCAGCATATCATCCGCAGCA
 641    ---------+---------+---------+---------+---------+---------+---------+---------+
        TTTGGCTGGCCATCGCGTCGTTAACCAGACTCGTACCGTCCATCGTCGCGTTACTAAGACCGTCGTATAGTAGGCGTCGT
        K__P__T__G__S__A__A__I__G__L__S__M__A__G__S__S__A__M__I__L__A__A__Y__H__P__Q__Q_

        GTTTATCTATGCAGGTAGCCTGAGCGCACTGCTGGACCCGAGCCAGGGTATGGGTCCGAGCCTGATTGGTCTGGCAATGG
 721    ---------+---------+---------+---------+---------+---------+---------+---------+
        CAAATAGATACGTCCATCGGACTCGCGTGACGACCTGGGCTCGGTCCCATACCCAGGCTCGGACTAACCAGACCGTTACC
        _F__I__Y__A__G__S__L__S__A__L__L__D__P__S__Q__G__M__G__P__S__L__I__G__L__A__M__G

        GTGATGCCGGTGGTTATAAAGCAGCAGATATGTGGGGTCCGAGCAGCGATCCGGCATGGGAACGTAATGATCCGACCCAG
 801    ---------+---------+---------+---------+---------+---------+---------+---------+
        CACTACGGCCACCAATATTTCGTCGTCTATACACCCCAGGCTCGTCGCTAGGCCGTACCCTTGCATTACTAGGCTGGGTC
        __D__A__G__G__Y__K__A__A__D__M__W__G__P__S__S__D__P__A__W__E__R__N__D__P__T__Q__

        CAGATTCCGAAACTGGTTGCAAATAATACCCGTCTGTGGGTTTATTGTGGTAATGGCACCCCGAATGAACTGGGTGGTGC
 881    ---------+---------+---------+---------+---------+---------+---------+---------+
        GTCTAAGGCTTTGACCAACGTTTATTATGGGCAGACACCCAAATAACACCATTACCGTGGGGCTTACTTGACCCACCACG
        Q__I__P__K__L__V__A__N__N__T__R__L__W__V__Y__C__G__N__G__T__P__N__E__L__G__G__A_

        AAATATTCCGGCAGAATTTCTGGAAAATTTTGTGCGTAGCAGCAACCTGAAATTCCAGGATGCATATAATGCAGCCGGTG
 961    ---------+---------+---------+---------+---------+---------+---------+---------+
        TTTATAAGGCCGTCTTAAAGACCTTTTAAAACACGCATCGTCGTTGGACTTTAAGGTCCTACGTATATTACGTCGGCCAC
        _N__I__P__A__E__F__L__E__N__F__V__R__S__S__N__L__K__F__Q__D__A__Y__N__A__A__G__G

        GTCATAATGCCGTTTTTAACTTTCCGCCTAATGGCACCCATAGCTGGGAATATTGGGGTGCACAGCTGAATGCAATGAAA
1041    ---------+---------+---------+---------+---------+---------+---------+---------+
        CAGTATTACGGCAAAAATTGAAAGGCGGATTACCGTGGGTATCGACCCTTATAACCCCACGTGTCGACTTACGTTACTTT
        __H__N__A__V__F__N__F__P__P__N__G__T__H__S__W__E__Y__W__G__A__Q__L__N__A__M__K__

                                         EcoRI
        GGTGATCTGCAGAGCAGCCTGGGTGCAGGTTAAGAATTC
1121    ---------+---------+---------+---------  1159
        CCACTAGACGTCTCGTCGGACCCACGTCCAATTCTTAAG
        G__D__L__Q__S__S__L__G__A__G__*__
```

Legend Figure S1. Nucleotide sequences of the synthetic genes coding for TB10.4, Ag85B and "*full*" proteins and corresponding amino acid sequences. Restriction sites used for cloning are indicated. Genes are subcloned in pET32 and pColdI plasmids in frame with the plasmid sequence containing the codon for the N-terminal Met (see text for details).

Figure S2

Trx-TB10.4 (261 aa)

MSDKIIHLTDDSFDTDVLKADGAILVDFWAEWCGPCKMIAPILDEIADEYQGKLTVAKLNIDQNPGTAPKYGIRGIPT
LLLFKNGEVAATKVGALSKGQLKEFLDANLAGSGSGHM*HHHHHHSSGLVPRGSGMKETAAAKFERQHMDSPDLGTDDD*
*DKAMAISDP***MSQIMYNYPAMLGHAGDMAGYAGTLQSLGAEIAVEQAALQSAWQGDTGITYQAWQAQWNQAMEDLVRAY**
HAMSSTHEANTMAMMARDTAEAAKWGG

TB10.4 (103 aa)

*AMAISDP***MSQIMYNYPAMLGHAGDMAGYAGTLQSLGAEIAVEQAALQSAWQGDTGITYQAWQAQWNQAMEDLVRAYHA**
MSSTHEANTMAMMARDTAEAAKWGG

Trx-Ag85B (450 aa)

MSDKIIHLTDDSFDTDVLKADGAILVDFWAEWCGPCKMIAPILDEIADEYQGKLTVAKLNIDQNPGTAPKYGIRGIPT
LLLFKNGEVAATKVGALSKGQLKEFLDANLAGSGSGHM*HHHHHHSSGLVPRGSGMKETAAAKFERQHMDSPDLGTDDD*
*DKAMAISDP***FSRPGLPVEYLQVPSPSMGRDIKVQFQSGGNNSPAVYLLDGLRAQDDYNGWDINTPAFEWYYQSGLSIV**
MPVGGQSSFYSDWYSPACGKAGCQTYKWETFLTSELPQWLSANRAVKPTGSAAIGLSMAGSSAMILAAYHPQQFIYAG
SLSALLDPSQGMGPSLIGLAMGDAGGYKAADMWGPSSDPAWERNDPTQQIPKLVANNTRLWVYCGNGTPNELGGANIP
AEFLENFVRSSNLKFQDAYNAAGGHNAVFNFPPNGTHSWEYWGAQLNAMKGDLQSSLGAG

Ag85B (292 aa)

*AMAISDP***FSRPGLPVEYLQVPSPSMGRDIKVQFQSGGNNSPAVYLLDGLRAQDDYNGWDINTPAFEWYYQSGLSIVMP**
VGGQSSFYSDWYSPACGKAGCQTYKWETFLTSELPQWLSANRAVKPTGSAAIGLSMAGSSAMILAAYHPQQFIYAGSL
SALLDPSQGMGPSLIGLAMGDAGGYKAADMWGPSSDPAWERNDPTQQIPKLVANNTRLWVYCGNGTPNELGGANIPAE
FLENFVRSSNLKFQDAYNAAGGHNAVFNFPPNGTHSWEYWGAQLNAMKGDLQSSLGAG

Trx-*full* (546 aa)

MSDKIIHLTDDSFDTDVLKADGAILVDFWAEWCGPCKMIAPILDEIADEYQGKLTVAKLNIDQNPGTAPKYGIRGIPT
LLLFKNGEVAATKVGALSKGQLKEFLDANLAGSGSGHM*HHHHHHSSGLVPRGSGMKETAAAKFERQHMDSPDLGTDDD*
*DKAMAISDP***MSQIMYNYPAMLGHAGDMAGYAGTLQSLGAEIAVEQAALQSAWQGDTGITYQAWQAQWNQAMEDLVRAY**
HAMSSTHEANTMAMMARDTAEAAKWGGFSRPGLPVEYLQVPSPSMGRDIKVQFQSGGNNSPAVYLLDGLRAQDDYNGW
DINTPAFEWYYQSGLSIVMPVGGQSSFYSDWYSPACGKAGCQTYKWETFLTSELPQWLSANRAVKPTGSAAIGLSMAG
SSAMILAAYHPQQFIYAGSLSALLDPSQGMGPSLIGLAMGDAGGYKAADMWGPSSDPAWERNDPTQQIPKLVANNTRL
WVYCGNGTPNELGGANIPAEFLENFVRSSNLKFQDAYNAAGGHNAVFNFPPNGTHSWEYWGAQLNAMKGDLQSSLGAG

His-*full*1 (414 aa)

MNHKV*HHHHHH*IEGRHMELGT*DDDDK*AMAISDP**MSQIMYNYPAMLGHAGDMAGYAGTLQSLGAEIAVEQAALQSAWQG**
DTGITYQAWQAQWNQAMEDLVRAYHAMSSTHEANTMAMMARDTAEAAKWGGFSRPGLPVEYLQVPSPSMGRDIKVQFQ
SGGNNSPAVYLLDGLRAQDDYNGWDINTPAFEWYYQSGLSIVMPVGGQSSFYSDWYSPACGKAGCQTYKWETFLTSEL
PQWLSANRAVKPTGSAAIGLSMAGSSAMILAAYHPQQFIYAGSLSALLDPSQGMGPSLIGLAMGDAGGYKAADMWGPS
SDPAWERNDPTQQIPKLVANNTRLWVYCGNGTPNELGGANIPAEFLENFVRSSNLKFQDAYNAAGGHNAVFNFPPNGT
HSWEYWGAQLNAMKGDLQSSLGAG

*full*1 (388 aa)

AMAISDP**MSQIMYNYPAMLGHAGDMAGYAGTLQSLGAEIAVEQAALQSAWQGDTGITYQAWQAQWNQAMEDLVRAYHA**
MSSTHEANTMAMMARDTAEAAKWGGFSRPGLPVEYLQVPSPSMGRDIKVQFQSGGNNSPAVYLLDGLRAQDDYNGWDI
NTPAFEWYYQSGLSIVMPVGGQSSFYSDWYSPACGKAGCQTYKWETFLTSELPQWLSANRAVKPTGSAAIGLSMAGSS
AMILAAYHPQQFIYAGSLSALLDPSQGMGPSLIGLAMGDAGGYKAADMWGPSSDPAWERNDPTQQIPKLVANNTRLWV
YCGNGTPNELGGANIPAEFLENFVRSSNLKFQDAYNAAGGHNAVFNFPPNGTHSWEYWGAQLNAMKGDLQSSLGAG

His-*full*2 (397 aa)

MNHKV*HHHHHHIEGR*H**MSQIMYNYPAMLGHAGDMAGYAGTLQSLGAEIAVEQAALQSAWQGDTGITYQAWQAQWNQAM**
EDLVRAYHAMSSTHEANTMAMMARDTAEAAKWGGFSRPGLPVEYLQVPSPSMGRDIKVQFQSGGNNSPAVYLLDGLRA
QDDYNGWDINTPAFEWYYQSGLSIVMPVGGQSSFYSDWYSPACGKAGCQTYKWETFLTSELPQWLSANRAVKPTGSAA
IGLSMAGSSAMILAAYHPQQFIYAGSLSALLDPSQGMGPSLIGLAMGDAGGYKAADMWGPSSDPAWERNDPTQQIPKL
VANNTRLWVYCGNGTPNELGGANIPAEFLENFVRSSNLKFQDAYNAAGGHNAVFNFPPNGTHSWEYWGAQLNAMKGDL
QSSLGAG

Figure S2. Amino acid sequences of the different recombinant proteins as produced in *E. coli* upon cloning in pET32b plasmid (Trx-TB10.4, Trx-Ag85B and Trx-*full*) or in pColdI plasmid (His-*full1* and His-*full2*) and as obtained upon proteolytic cleavage (TB10.4, Ag85B and *full*1). TB10.4 and Ag85B sequences encoded by the synthetic genes are evidenced in bold (TB10.4) and in bold and underlined (Ag85B). His_6-tag and EK (DDDDK) cleavage sites (both arising from the cloning procedures) are in italics and underlined.
