# Supplementary material for: Optimizing Escherichia coli as a protein expression platform to produce Mycobacterium tuberculosis immunogenic proteins
Source: Microb Cell Fact. 2013 Nov 19;12:115. doi: 10.1186/1475-2859-12-115 (PMC4225511; doi:10.1186/1475-2859-12-115)
Supplement: Additional file 2 — Expression and purification of antigenic proteins. [file 1475-2859-12-115-S2.pdf]

## Additional File 2. Expression and purification of antigenic proteins.

**Figure S3**

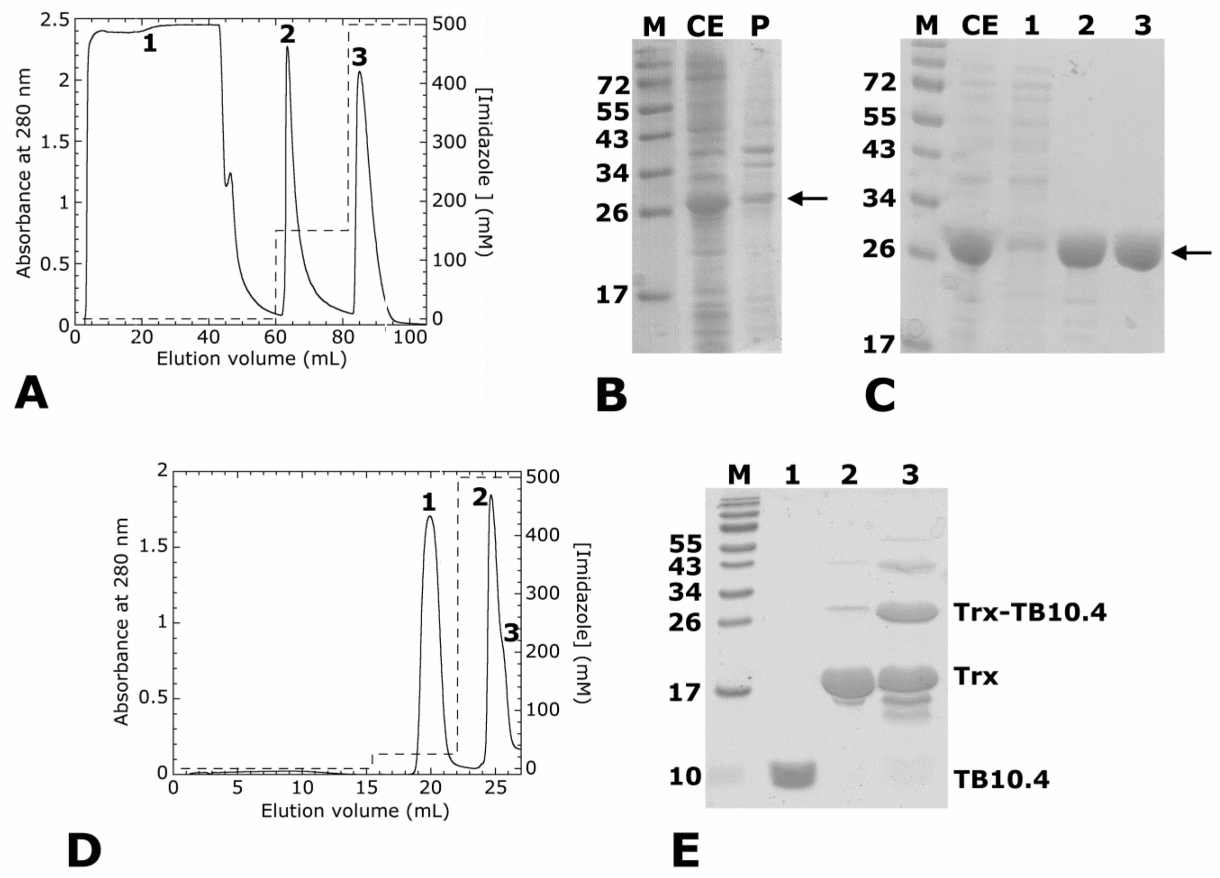


**Figure S3.** Purification of Trx-TB10.4 chimeric protein from *E. coli* (BL21)DE3 cells (A, B and C) and separation of the proteolytic products Trx and TB10.4 after cleavage with enterokinase (D and E). *E. coli* (BL21)DE3 cells were grown in LB medium at 37 °C; protein production was induced adding 0.1 mM IPTG when $OD_{600nm}$ reached 0.8; cells were collected after 16 hours of additional growth at 18 °C. Starting material: 4.8 g of *E. coli* cell paste (wet weight). Panel A: elution profile of purification of Trx-TB10.4 chimeric protein by nickel-affinity chromatography on HiTrap Chelating column. Panel B: SDS-PAGE analysis of soluble (CE, crude extract) and insoluble (P, pellet) fractions obtained after cell disruption and centrifugation; an amount corresponding to 50 µL of cultivation broth was loaded. The arrow indicates the position of the Trx-TB10.4 chimeric protein. Panel C: SDS-PAGE analysis of selected fractions from chromatographic separation reported in panel A. CE: crude (soluble) extract; 1: flow-through; 2: fraction eluted at 150 mM imidazole; 3: fraction eluted at 500 mM imidazole (pure Trx-TB10.4, indicated by an arrow). Panel

D: elution profile of the separation on HiTrap Chelating column of Trx and TB10.4 following the proteolytic cleavage. Cleavage conditions: 20 mg of Trx-TB10.4 chimera were incubated for 6 hours at 25 °C with 40 U of recombinant enterokinase. Panel E: SDS-PAGE analysis of the fractions obtained from purification reported in panel D. 1: fraction eluted at 25 mM imidazole (pure TB10.4); 2-3: fractions eluted at 500 mM imidazole (Trx and not cleaved Trx-TB10.4). M: molecular mass markers (indicated in kDa on the left side of panels B, C and D).

**Figure S4**

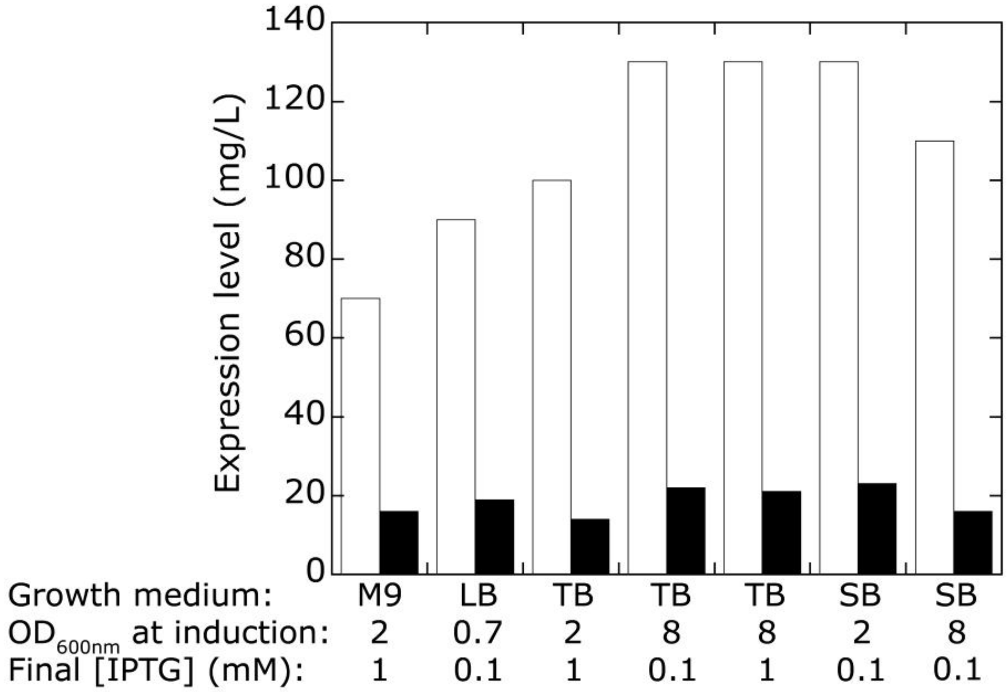


**Figure S4.** Expression level of Trx-Ag85B chimeric protein (expressed as milligram of protein per liter of cultivation broth) under different conditions (reported below bars) and as determined by Western blot analysis using an anti-His-tag monoclonal antibody. In all cases, following IPTG addition, cells were grown at 18 °C and collected after 4 hours. White bars: overall expressed protein (from whole cell extract); black bars: soluble expressed protein (from crude soluble extract).

**Figure S5**

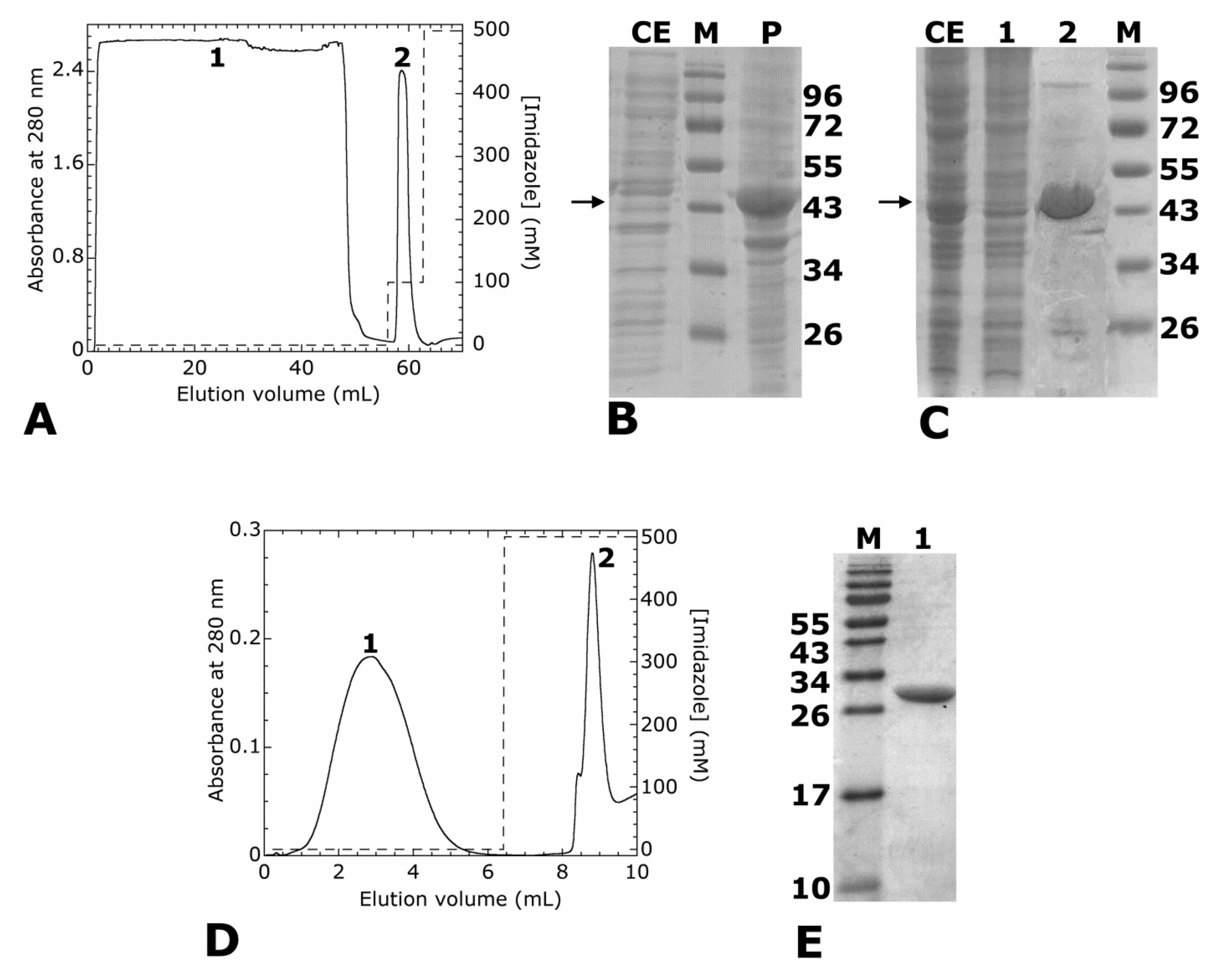


**Legend Figure S5**. Purification of Trx-Ag85B chimeric protein from *E. coli* BL21(DE3) cells (A, B and C) and separation of the proteolytic products Trx and Ag85B after cleavage with enterokinase (D and E). *E. coli* cells were grown in SB medium at 37 °C. Protein production was induced adding 0.1 mM IPTG when $OD_{600nm}$ reached 2; cells were collected after 16 hours of additional growth at 18 °C. Starting material: 10 g of *E. coli* cell paste (wet weight). Panel A: elution profile of purification of Trx-Ag85B chimeric protein by nickel-affinity chromatography on HiTrap Chelating column. Panel B: SDS-PAGE analysis of soluble (CE, crude extract) and insoluble (P, pellet) fractions obtained after cell disruption and centrifugation; an amount corresponding to 50 µL of cultivation broth was loaded. The arrow indicates the position of the Trx-Ag85B chimeric protein. Panel C: SDS-PAGE analysis of the fractions as in panel A. CE: crude (soluble) extract; 1: flow-through; 2: fraction eluted at 100 mM imidazole (pure Trx-Ag85B, indicated by the arrow). Panel D: elution profile of the separation on HiTrap Chelating column of

Trx and Ag85B following the proteolytic cleavage. Cleavage conditions: 16.5 mg of Trx-Ag85B chimera were incubated for 16 hours at 20 °C with 50 U of recombinant enterokinase. Panel E: SDS-PAGE analysis of the fraction containing the pure Ag85B (1, flow-through). M: molecular mass markers (indicated in kDa on the side of panels B, C and E).

**Figure S6**

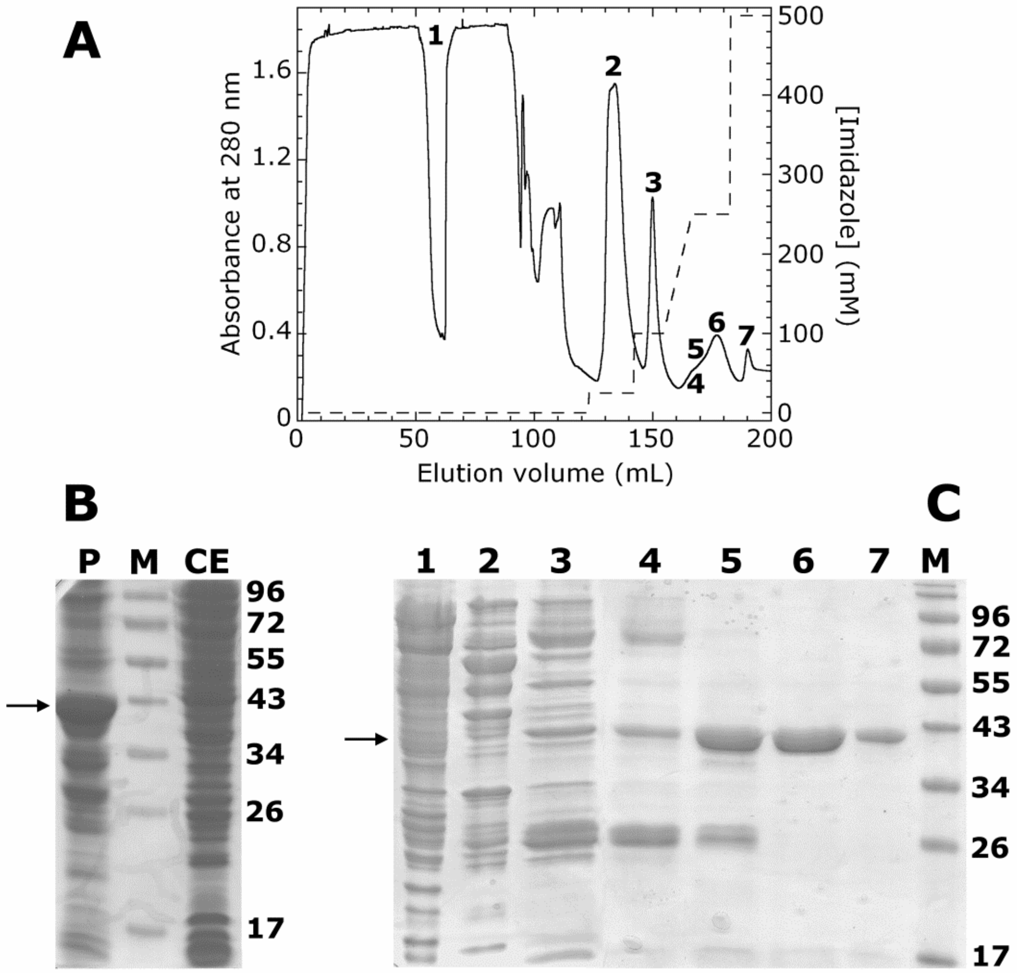


**Legend Figure S6.** Purification of His-*full*2 chimeric protein from *E. coli* BL21(DE3) cells. *E. coli* cells were grown in 3 L bioreactor in SB/NaCl medium at 37 °C. Protein production was induced by adding 0.1 mM IPTG and 25 g/L NaCl when $OD_{600nm}$ reached 2; cells were collected after additional 16 hours of growth at 15 °C. Starting material: 13 g of *E. coli* cell paste (wet weight). Panel A: elution profile of purification of His-*full*2 chimeric protein by chromatography on HiTrap Chelating column. Panel B: SDS-PAGE analysis of soluble (CE, crude extract) and insoluble (P, pellet) fractions obtained after cell disruption and centrifugation; an amount corresponding to 50 µL of cultivation broth was loaded. The arrow indicates the position of the His-*full2* protein. Panel C: SDS-PAGE analysis of fractions as in panel A. 1: flow-through; 2: fraction eluted at 25 mM imidazole; 3: fraction eluted at 100 mM imidazole; 4 – 6: fractions eluted during the linear gradient from 100 to 250 mM imidazole; 7: fraction eluted at 500 mM imidazole. Fractions 6 and 7 contain

pure His-*full*2 (indicated by the arrow). M: molecular mass markers (indicated in kDa on the side of the panel B and C).

**Figure S7**

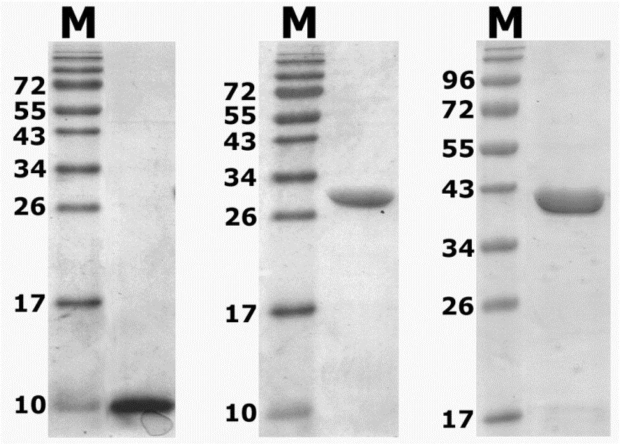


**Legend Figure S7.** SDS-PAGE analysis of the pure, mature proteins. Left panel: TB10.4; central panel: Ag85B; right panel: His-*full*2. M: molecular mass markers (indicated in kDa on the left side of each panel).
