# Supplementary material for: Optimizing Escherichia coli as a protein expression platform to produce Mycobacterium tuberculosis immunogenic proteins
Source: Microb Cell Fact. 2013 Nov 19;12:115. doi: 10.1186/1475-2859-12-115 (PMC4225511; doi:10.1186/1475-2859-12-115)
Supplement: Additional file 3 — Immunological assays. [file 1475-2859-12-115-S3.pdf]

Additional File 3. Immunological assays.

Figure S8

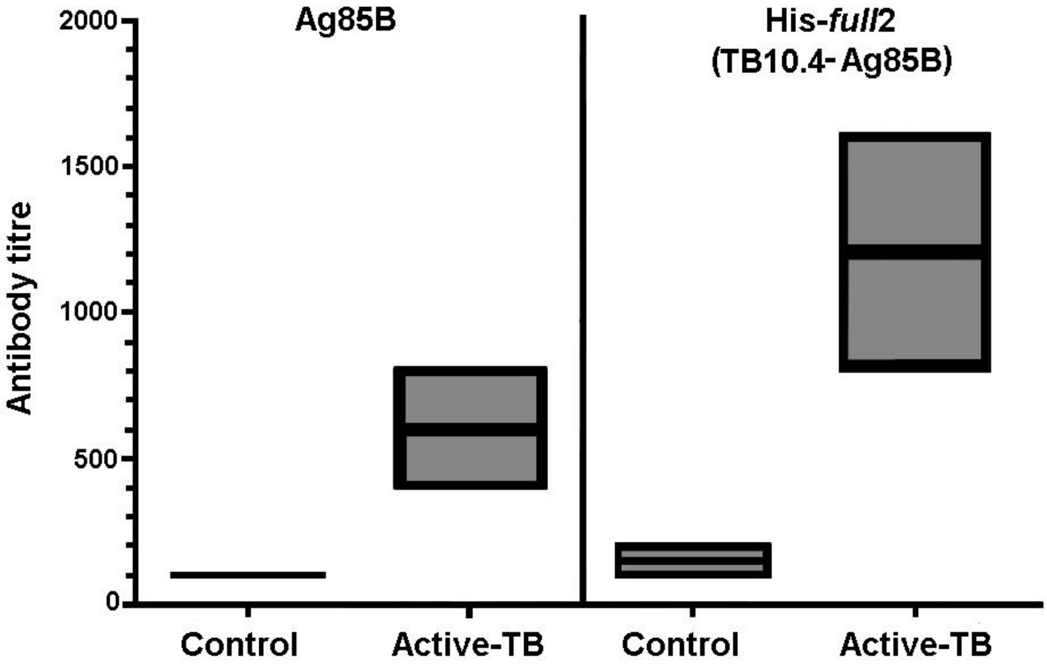

Legend Figure S8. Analysis of the antibody titre against Ag85B and His-*full*2 (TB10.4-Ag85B) proteins. For each control and active TB serum, the inverse of the last dilution still showing an OD double than the control without sera (antibody titre) against the Ag85B and the His-*full*2 proteins is reported; the value is depicted as minimum and maximum of the observed reactivity. End final point test has been performed starting from 1:50 dilution.

Figure S9

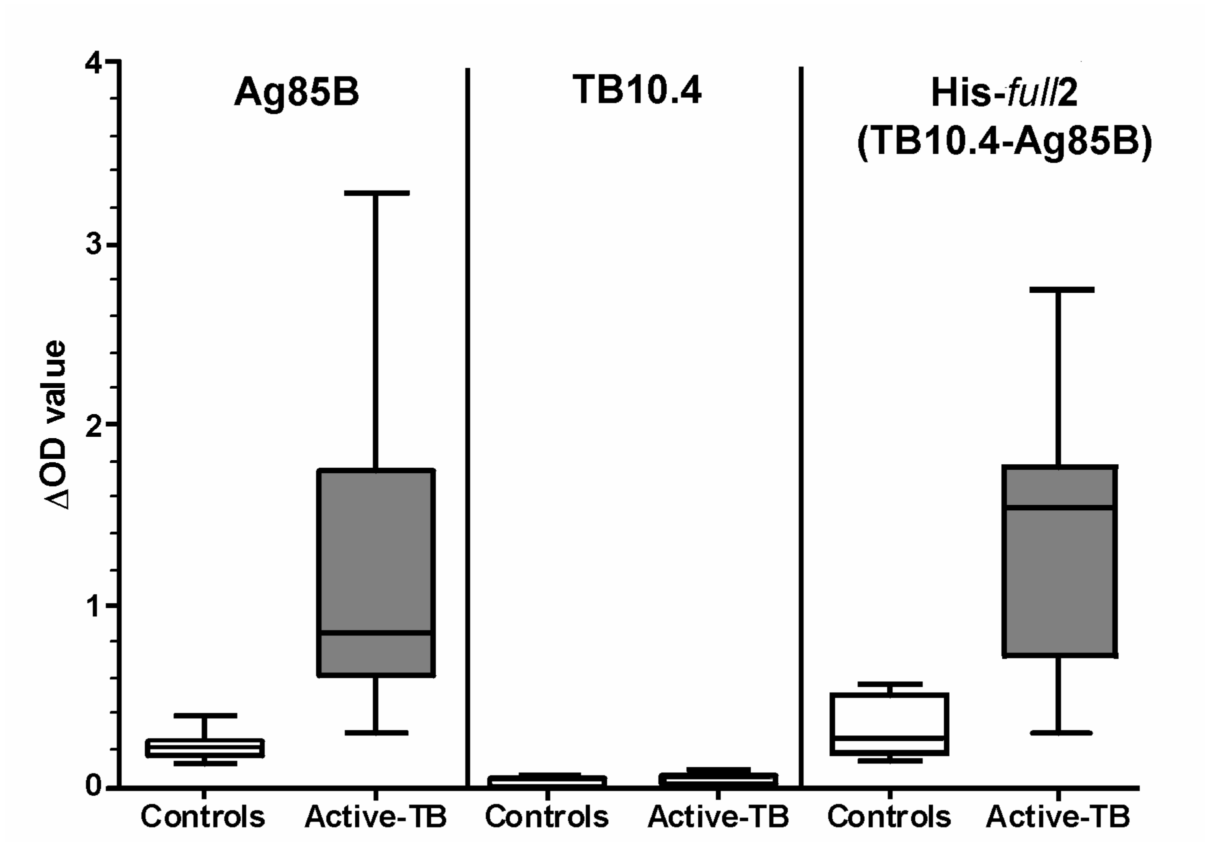

Legend Figure S9. Ag85B, TB10.4 and His-*full*2 (TB10.4-Ag85B) proteins (200 ng each) were tested on ELISA plate vs. active-TB and control sera at 1:100 dilution. The reactivity is reported as difference of the OD (ΔOD) observed for the active and control sera in presence and in absence of serum, reporting the median, 25-75 percentile range (box), and minimum and maximum value (line) observed for each patient.
